# Fungal Elevational Rapoport pattern from a High Mountain in Japan

**DOI:** 10.1038/s41598-019-43025-9

**Published:** 2019-04-25

**Authors:** Matthew Chidozie Ogwu, Koichi Takahashi, Ke Dong, Ho-Kyung Song, Itumeleng Moroenyane, Bruce Waldman, Jonathan M. Adams

**Affiliations:** 1 School of Biological Sciences, Seoul National University, 1 Gwanak-ro, Gwanak-gu, Seoul, 08826 Republic of Korea; 2 Department of Plant Biology and Biotechnology, University of Benin, PMB 1154 Ugbowo, Benin City, Edo State Nigeria; 3 Department of Biology, Faculty of Science, Shinshu University, Nagano, 390-8621 Japan; 4 Department of Life Sciences, Kyonggi University, Suwon, 443-760 Republic of Korea; 5 Institut National de la Recherche Scientifique, Centre INRS-Institut Armand-Frappier, 531 Boulevard de Prairies, Laval, Quebec, H7V 1B7 Canada; 6 Department of Integrative Biology, Oklahoma State University, 501 Life Sciences West, Stillwater, Oklahoma, 74078 USA; 7 Division of Soils and Agrifood, School of Water, Energy and Environment, Building 52a, Cranfield University, Bedfordshire, MK43 0AL United Kingdom

**Keywords:** Fungal ecology, Biodiversity

## Abstract

Little is known of how fungal distribution ranges vary with elevation. We studied fungal diversity and community composition from 740 to 2940 m above sea level on Mt. Norikura, Japan, sequencing the ITS2 region. There was a clear trend, repeated across each of the fungal phyla (Basidiomycota, Ascomycota, Zygomycota, Chytridomycota and Glomeromycota), and across the whole fungal community combined, towards an increased elevational range of higher elevation OTUs, conforming to the elevational Rapoport pattern. It appears that fungi from higher elevation environments are more generalized ecologically, at least in terms of climate-related gradients. These findings add to the picture from latitudinal studies of fungal ranges, which also suggest that the classic Rapoport Rule (broader ranges at higher latitudes) applies on a geographical scale. However, there was no mid-elevation maximum in diversity in any of the phyla studied, and different diversity trends for the different phyla, when different diversity indices were used. In terms of functional guilds, on Norikura there were trends towards increased saprotrophism (Zygomycota), symbiotrophism (Basidiomycota), symbiotrophism and saprotrophism (Ascomycota) and pathotrophism (Chytridiomycota) with elevation. The causes of each of these trends require further investigation from an ecological and evolutionary viewpoint.

## Introduction

Elevational patterns of organisms have long been a major focus in ecology^1–6^. In the past few years, this has included work on elevational patterns in bacterial diversity and community structure^7–13^. However, there has been surprisingly little work on elevational trends in fungal communities using advanced metagenetic methods, despite interest in general fungal distributions^14^. Various elevational studies of soil fungi have been published – including the Himalayan Mountains (Nepal), Colorado Rocky Mountain (USA), Mountains Milla and Segrilla (Tibet), Central Veracruz (Mexico), Mt. Fuji (Japan) and Mt. Kinabalu (Malaysia)^15–20^. However, despite their wide geographical scatter, these studies mostly focused on specific phylogenetically or ecologically defined fungal subgroups such as Chytridiomycota, arbuscular mycorrhizal (AM) fungi, macromycetes and ectomycorrhizal (EcM) fungi.

In the present study, we were particularly interested in investigating a pattern proposed on the basis of Rapoport’s Rule, which states that organisms at higher latitudes have broader distribution ranges due to the more variable temperature conditions in higher latitudes^21–24^. The existence of a parallel range extent pattern with elevation has been predicted, with organisms from higher elevations expected to have broader distribution ranges than those at lower elevations: this has been termed the Rapoport’s elevation (RE) gradients^24,25^. According to Stevens^24^ and McCain & Knight^26^, the RE pattern is a product of the increasing ecological tolerance range necessary for the survival of organisms at higher elevations, due to a parallel increase in environmental variability, and resulting variability in ecosystem fluxes at higher elevations. Amongst studies published so far, mostly on larger organisms, some have supported the existence of a RE pattern^25,27,28^, while others have not^29–31^. In a recent study on Mt. Norikura, Dong *et al*.^32^ found an elevational RE pattern in soil nematodes. None of the previously published studies on elevational trends in fungal subgroups has covered the subject of RE.

We were also interested in understanding how fungal diversity and community composition varied along elevational gradients. Many studies – of various types of organism - have found mid-elevational maximum in diversity, which has been attributed to various causes^32^. Miyamoto *et al*.^18^ found a mid-elevation maximum in diversity of EcM fungi on Mt. Fuji, which might relate to the abundant EcM-dependent perennial shrubs such as *Pyrola* and *Orthilia* spp. on the mountain. We were also interested in studying whether mid-elevation EcM diversity maxima would also be found across the fungal community on Mt. Norikura. Mt. Norikura has a very similar climate to Mt. Fuji, the two mountains being only about 240 Km apart, and of similar elevation. However, they differ in terms of geological history, Norikura is composed of andesitic rocks in contrast to Fuji’s basaltic origin. While the vegetation cover of the upper half of Fuji was largely destroyed several thousand years ago by volcanic eruptions^33^ and has not yet returned, the vegetation of Norikura has not been destroyed in the last 20,000 years^34^ and extends to considerably higher elevations, apparently being limited more by climate than soil development. Given the similar set of climatic circumstances but different history of these two mountains, it is of interest to test whether similar diversity patterns occur.

## Results

### Community composition

A total of 2,793,205 quality fungal sequences (containing 442,942 unique sequences) with an average length of 333 base pairs were obtained from the 55 samples, which were classified into 42,732 operational taxonomic units (OTU’s) at a 99% similarity level. Despite this level of coverage, the lack of asymptotes in the rarefaction curves (Supplementary Fig. 1) suggests that considerable fungal diversity remains un-sampled, as is typical of analyses of microbial diversity.

**Figure 1:**
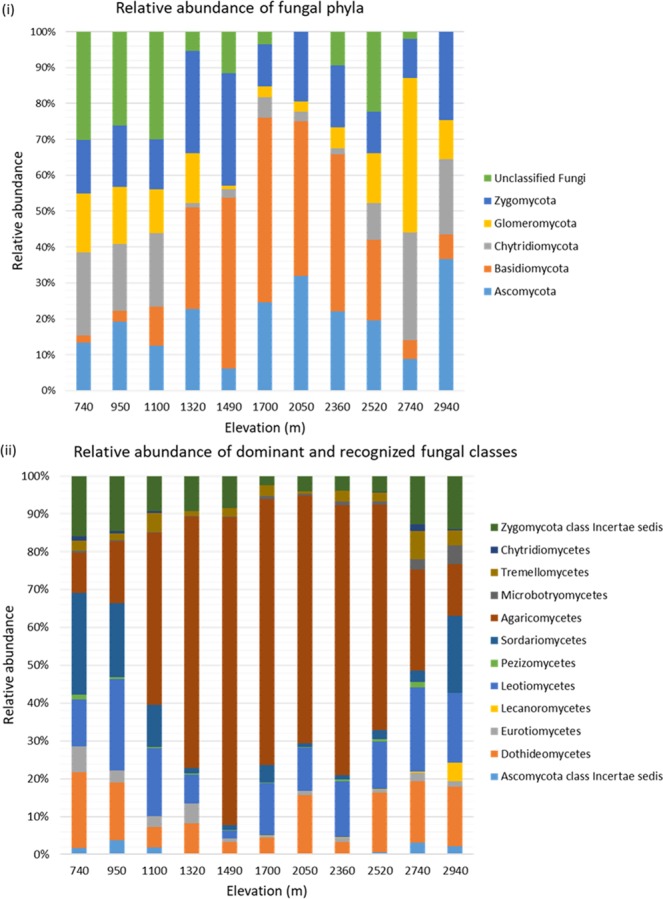
Relative average abundances of fungal phyla (i) and dominant and recognized classes (ii) at different elevational isoclines.

We found six fungal phyla on Norikura (Fig. 1), which showed significantly different trends with p < 0.05 (Supplementary Table 1). The mid-elevations were dominated by Basidiomycota and to a lesser degree by Ascomycota and then Zygomycota. In contrast, Ascomycota, Chytridiomycota, and Glomeromycota dominated higher elevations with significant presence of Zygomycota at lower elevations (Fig. 1i). The most abundant classes were Agaricomycetes, Sordariomycetes, Dothideomycetes, Leotiomycetes and Zygomycota class incertae sedis (Fig. 1ii).

**Figure 2:**
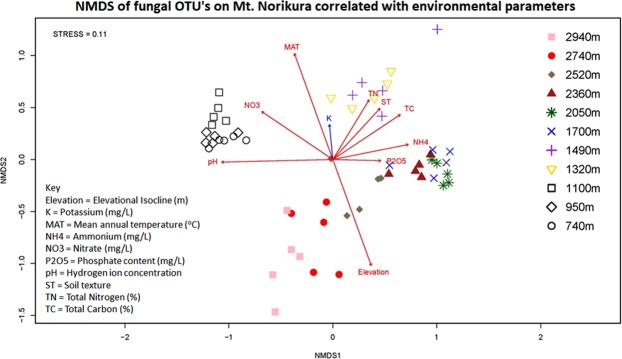
Compositional similarity relationship among samples on Mt. Norikura obtained from NMDS using Bray Curtis (of OTU’s) and fitted with Euclidean-based distance measure of environmental parameters.

A trend in community composition with elevation was identified using NMDS (Fig. 2). The NMDS plot revealed four main clusters, composed of one low elevation samples (740–1100 m a.s.l.), two mid-elevation samples (1320–2520 m a.s.l. and 2050–2360 m a.s.l.) and one high elevation samples (2740–2940 m a.s.l.), which clustered close together with few overlaps. Some overlapping was observed between high and mid-elevation samples (Fig. 2). Elevation had the strongest effect on community composition amongst high elevation samples, while the other environmental variables had greater effects amongst low and medium elevation samples. At the individual phylum level, the NMDS clustering pattern was similar to that of the overall fungal community in Ascomycota, Basidiomycota and Zygomycota (Supplementary Figs 2, 3 and 4). By contrast, Chytridiomycota, Glomeromycota and the *incertae cedis* category of ‘unclassified fungi’ showed no clear clustering pattern or trend in relation to any measured environmental factor (Supplementary Figs S5, S6 and S7). To corroborate our NMDS results, a one-way ANOSIM (analysis of similarities) test was used to test the relationship effects of elevation on community composition, and it showed that elevation had significant effect on observed fungal distribution (Supplementary Fig. 8; Global R = 0.915, Significant level = 0.1%).

### Rapoport’s elevation effect

**Figure 3:**
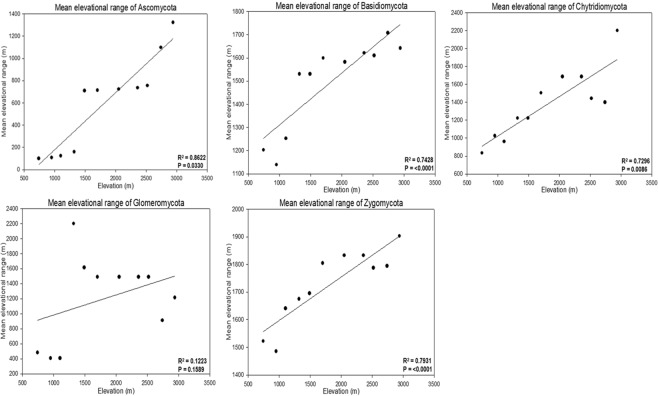
Elevation ranges in each of the major fungal phyla on Mt. Norikura. The mean range of OTUs in all phyla except Glomeromycota increased with elevation.

Elevational range was used to test the RE hypothesis according to Dong *et al*.^32^, Bhattarai & Vetaas^35^; (and see Supplementary Methods) revealing that the elevational range of individual phyla increased with elevation (Fig. 3). This suggested that mean species (defined as OTU) ranges increased proportionally with increasing elevation in the study (Fig. 3; Supplementary Fig. 9), i.e. fungal species found at low elevations had a narrower elevational range than those found at high elevations. When OTUs from all assigned fungal phyla were lumped together, there is also a clear increase in range extent with increasing elevation (Supplementary Fig. 9).

Amongst the various phyla, there is some variation in mean range extent, with OTUs of Basidiomycota and Zygomycota showing the broadest elevational ranges (Supplementary Fig. 10).

### Diversity patterns

**Figure 4:**
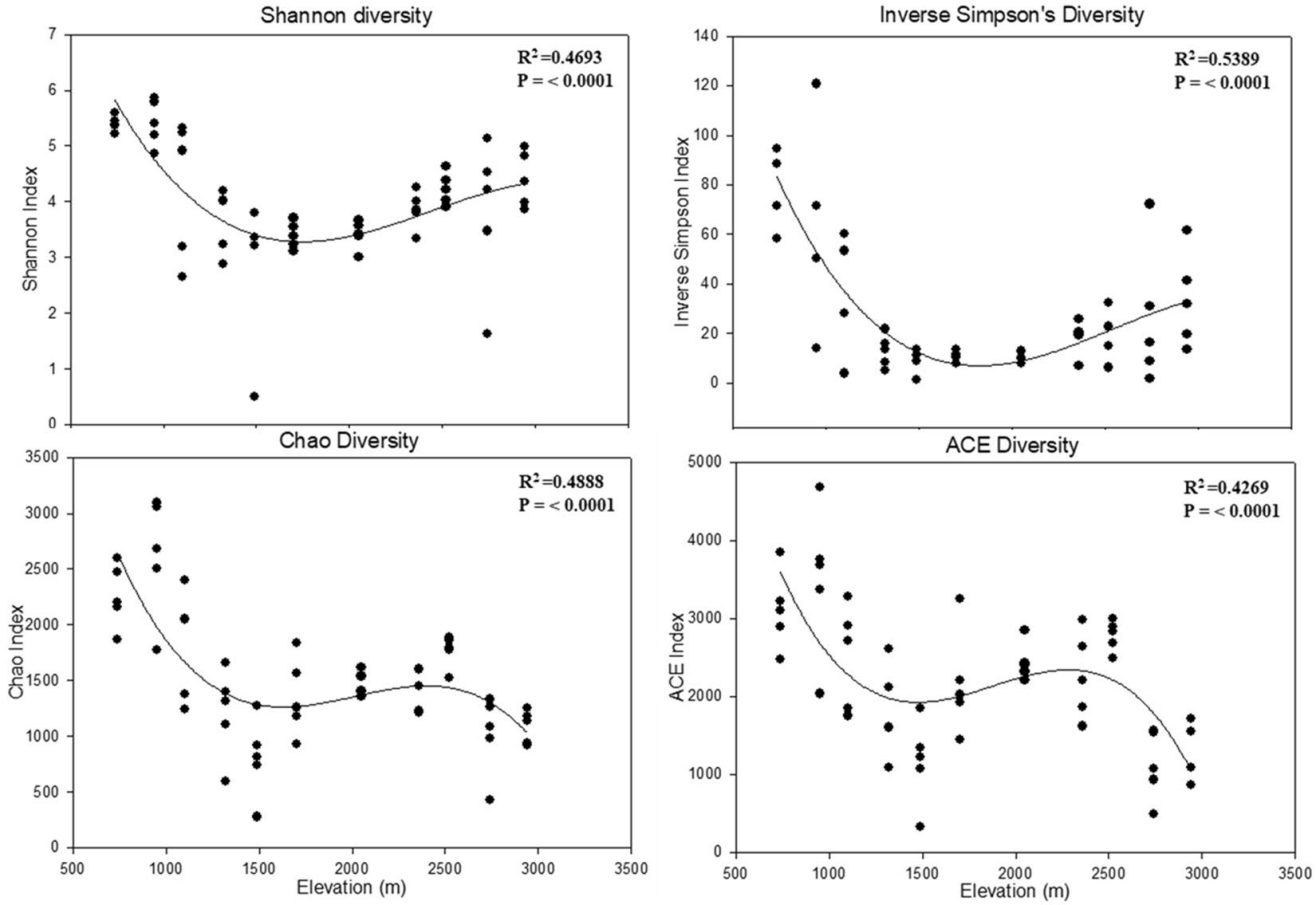
OTU diversity models for fungi assemblages along the elevation on Mt. Norikura. Linear, quadratic and cubic regression models were fitted to assess the relationship of elevation with diversity. Model selection was carried out based on adjusted R^2^and root mean square error. Only the best-fit linear and cubic regression were shown. Significance levels were less than 0.001, shown as ***. Adj R^2^ is coefficient of determination.

**Figure 5:**
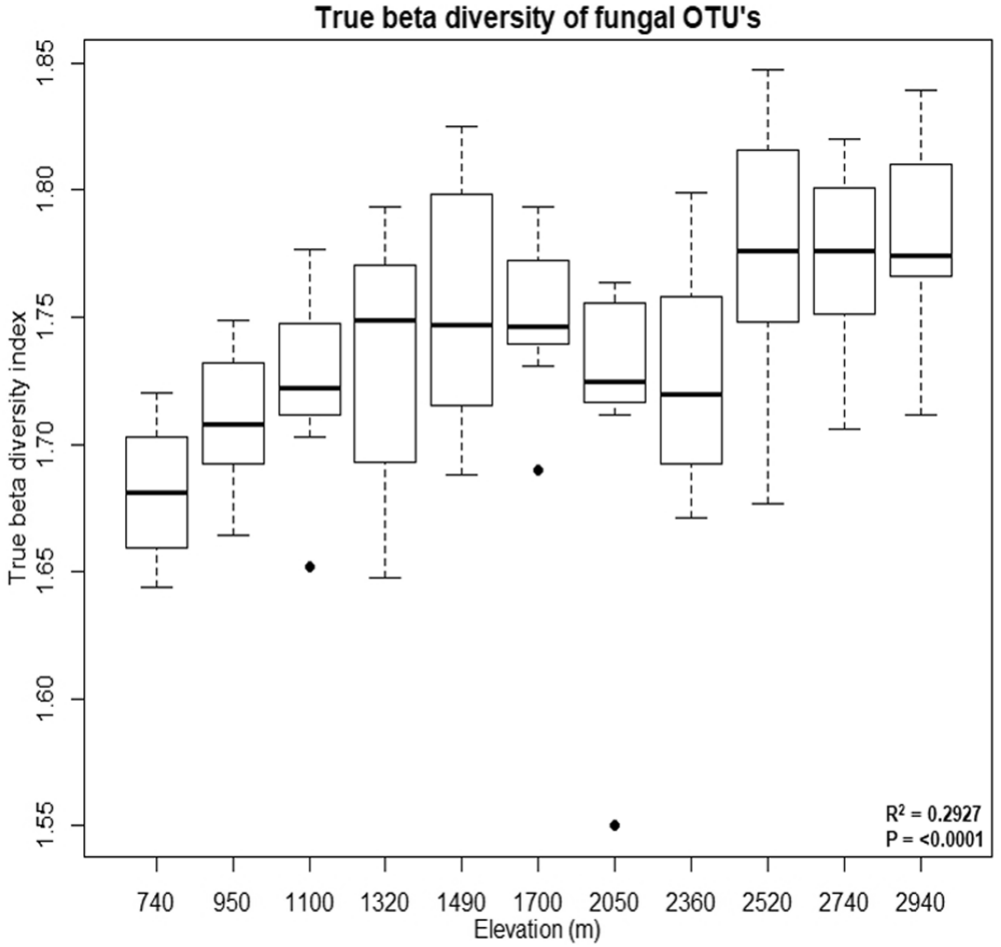
Beta diversity of fungi assemblages along the elevation on Mt. Norikura. Beta diversity increased with elevation on Mt. Norikura.

There was a negative correlation between overall alpha diversity and elevation on Mt. Norikura (Fig. 4). The different diversity models (Shannon, Inverse Simpson, Chao and abundance based coverage estimator (ACE)) showed a significant R^2^ and P value for mid-elevation minima for Shannon and Inverse Simpson but high elevation minima with Chao and ACE diversity indices. A similar pattern was observed regardless of the diversity model. The observed diversity patterns may have been caused by a complex – but unknown – combination of trends in the availability of different niches and ecotypes followed by the different taxa and trophic models. (Fig. 5). Overall, true β-diversity increased with elevation (Fig. 5).

### Functional categories

**Figure 6:**
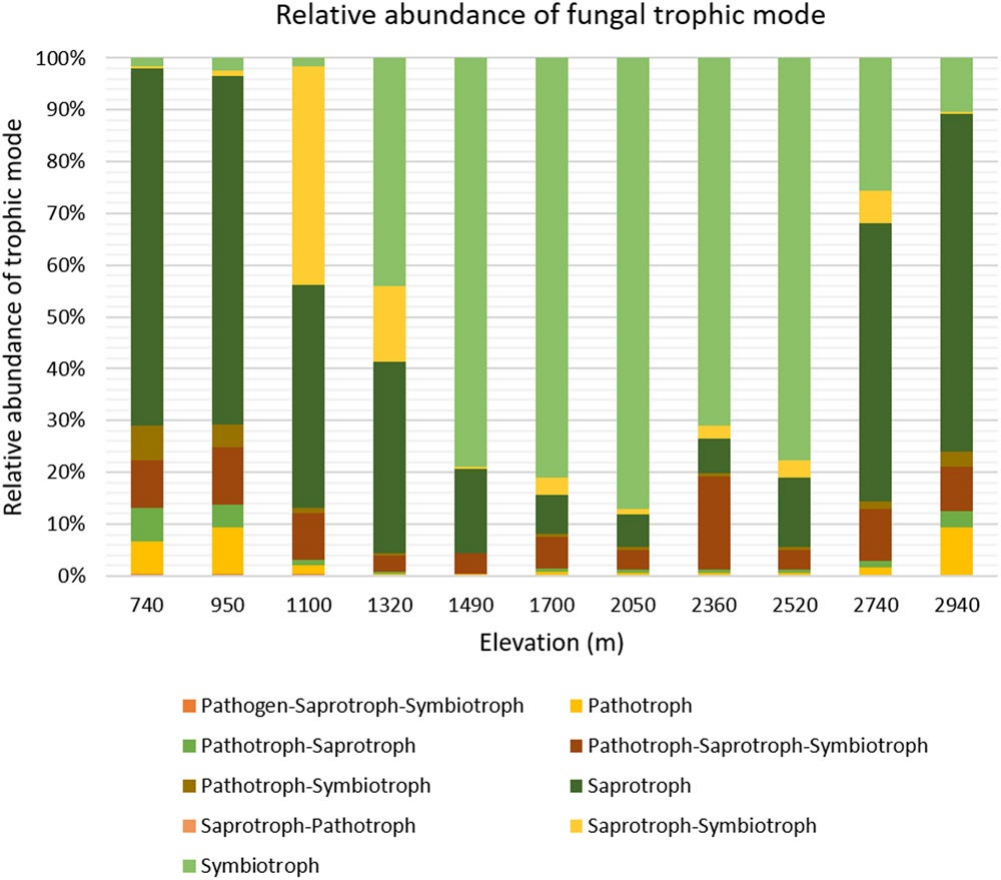
Fungal trophic mode along Mt. Norikura elevation, excluding OTUs unassigned at trophic level.

Nine recognized fungal trophic levels were found on Mt. Norikura including symbiotroph, saprotroph-symbiotroph, saprotroph-pathotroph, saprotroph, pathotroph-symbiotroph, pathotroph-saprotroph-symbiotroph, pathotroph-saprotroph, pathotroph, and pathotroph-saprotroph-symbiotroph. The unclassified trophic mode was eliminated from the analysis. The most abundant trophic mode differs along the elevational gradient. In the low and high elevation points, saprotrophic mode was the most dominant while in the mid-elevation point symbiotrophic mode was most abundant (Fig. 6).

The dominant trophic mode amongst Ascomycota was saprotrophic and to a lesser degree the symbiotrophic mode. The saprotrophic mode clearly dominates in low and mid-elevation point Ascomycota whereas symbiotrophic is dominant in the mid-elevation points (Supplementary Fig. 11). The dominant trophic mode varied to a greater degree in Basidiomycota (Supplementary Fig. 12). The low elevation is dominated by pathotrophic-saprotrophic-symbiotrophic as well as by pathotrophic and saprotroph-symbiotrophic mode, at the mid-elevation by symbiotrophic mode and high elevation by symbiotroph, pathotroph and pathotrophic-saprotrophic-symbiotrophic mode. Chytridiomycota on the other hand is dominated by pathotrophs as well as by pathotroph-saprotrophs and to a lesser degree by saprotrophs (Supplementary Fig. 13). The only trophic mode utilized by Glomeromycota is the symbiotrophic mode, and this phylum is relatively most abundant at the low, and high elevation points (Supplementary Fig. 14). Saprotrophic mode was the dominant mode for Zygomycota (Supplementary Fig. 15).

### Definition of ectomycorrhizal fungi on Mt. Norikura

A total of 79 ectomycorrhizal fungi (EcM) genera were identified according to the lists of Tedersoo *et al*.^36^ were found in Mt. Norikura, divided into three phyla; Ascomycota (13), Basidiomycota (64) and Zygomycota (2) (Supplementary Tables 2 and 3) respectively. This accounts for 9.5% of fungi genera found on Norikura. Amongst EcM, *Russula*, *Tylospora*, *Cenococcum*, *Cortinarius*, *Suillus*, *Amanita and Tricholoma* were the most abundant genera (Supplementary Fig. 16). The diversity, richness and RE effects conform to the overall community patterns for their respective phyla (Supplementary Fig. 17).

### Environmental characteristics and nestedness relationship

Principal component analysis (PCA) of environmental variables suggest two components account for more than 80% of observed variations (Supplementary Fig. 18). The environmental variables had directional effects on the fungi community along the Mt. Norikura elevation. Samples collected from within the same elevational isocline also clustered together.

**Figure 7:**
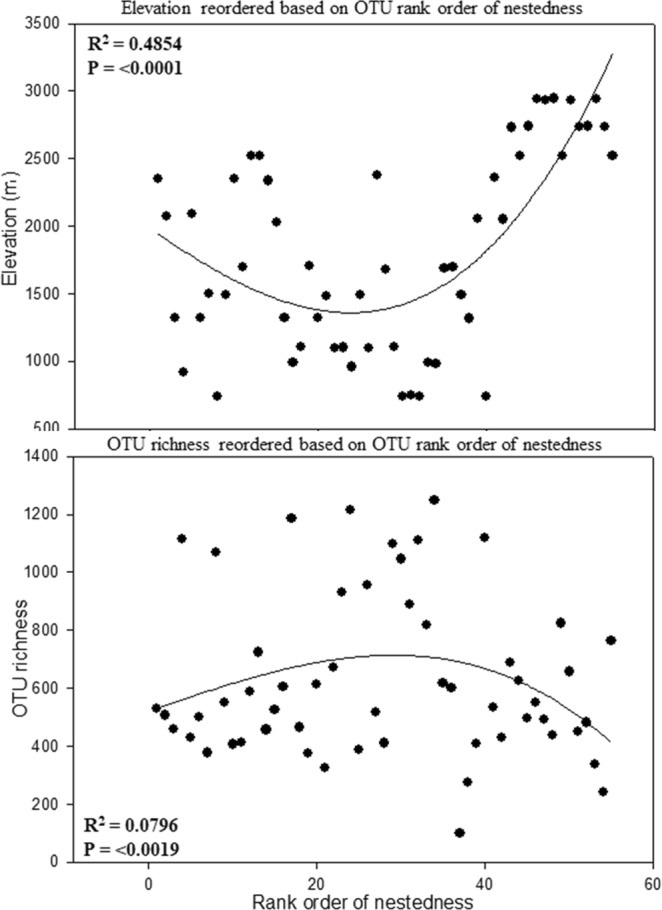
Rank order nestedness of fungal OTU’s on Mt. Norikurare ordered with elevation (m) and OTU richness. Rank order nestedness increased with elevation as OTU richness increased.

Best fit regression model of OTU nestedness was done with elevation and OTU richness (Fig. 7). There was a significant (R^2^ and P value) relationship between OTU nestedness with elevation and OTU richness. Elevation was nested above 1500 m a.s.l. while OTU richness was more nested below 700 m a.s.l. When the nested relationship was tested with all the measured environmental variables, significant R^2^ and P values were also obtained with different curve shapes (Supplementary Figs 19 and 20). Mean annual temperature had a clear humpback shape with most of the samples nested below 6 °C, below 40 mg/L nitrates, and below 5.5 pH.

## Discussion

### Increase in range extent with increasing elevation

The most striking pattern to emerge from this study was a clear increase in mean elevational range extent of fungal ‘proxy species’ (OTUs) (Fig. 3 and Supplementary Fig. 18). This is clearly in accordance with the expectations based on the RE pattern^22–24,37^ in contrast with McCain & Knight’s^26^ conclusion that Rapoport’s rule is not a useful tool for understanding community patterns on mountains – at least in plants, vertebrates and invertebrates.

The underlying causes of the RE trend we observed might include greater variability in temperature, precipitation and ecological productivity, or a lack of predictable resources. These factors might operate directly or indirectly through the food chain involving plants, detritus and animals – which are important food sources for soil fungi^5^. Variation in potential niche width may develop on evolutionary/geological time scales, and Veter *et al*.^38^ suggested that range extents are also filtered by differential extinction resulting from interspecific competition, to produce the observable variations in range extent. In this sense, the observed RE trend may be a product of present day ecological processes sorting the niches of fungi against a background of evolutionary time.

In this study, there were no mid-elevation diversity maxima, and thus the processes producing the RE pattern here are clearly distinct from those involved in producing mid-domain diversity maxima, despite previous attempts to link both patterns^39,40^.

In a broader context, there is also a clear Rapoport effect in the global/latitudinal ranges of fungi^14,41^, which broadly compliments the findings of our elevational study (Fig. 3, Supplementary Fig. 9). It is unclear if the factors producing these parallel patterns might be the same, and this is a subject, which requires further consideration through theory and observation.

Beyond evolutionary and community ecological processes, other underlying factors might have a significant role to play on elevational scales, in producing the observed elevational increase in range size: for example, the effect of airborne spore dispersal patterns on soil fungal community^42^. However, spores deposited from the air are expected to be a minor part of the DNA community found in a 10 cm-deep bulk soil sample. The observed pattern cannot be simply explained in terms of trophic relationships, as different fungal phyla showed quite different trends with elevation (Fig. 6). The trend we have found requires further investigation in terms of possible explanations, in terms of the ecology and evolution of niche width, and further sampling of elevational gradients elsewhere to understand how prevalent it is.

### Variation in relative abundance of trophic modes

There are clear elevation-related differences in the relative importance of different trophic modes on Norikura (Fig. 6, Supplementary Table 4). Such trends have been observed in other elevational studies on fungi, for example Geml *et al*.^20^ for Mt. Kinabalu using EcM fungi. Understanding such variations may require further study of the complex interactions and energy flows in the ecosystem, and how these are affected by the many factors that vary with elevation^43,44^. Variation in plant cover and litter (themselves affected by fungal activity and nutrient cycling), as well as interactions between different fungal groups, are likely to add to the complexity of fungal community patterns on Mt. Norikura^45^.

It is interesting that the mid-elevations of Mt. Norikura are dominated by symbiotrophic fungi (Fig. 6), mostly EcM fungi (Supplementary Fig. 16) and by Glomeromycota (Supplementary Fig. 14). This seems likely to relate to the dominance of EcM trees in the forest at these elevations^34^, especially *Betula*, alongside are range of other EcM-dependent Fagaceae, Pinaceae and Betulaceae.

### Diversity pattern

We found no evidence of consistent elevational diversity trends (Fig. 4), which might correlate with range extent. Unlike an earlier study by Miyamoto *et al*.^18^ on Mt. Fuji, we found no evidence of a mid-elevation diversity maximum in any of the groups of fungi we examined. Instead, our result was consistent with that reported by Liu *et al*.^46^, in showing some mid-elevation diversity minima and mid-elevation relative abundance maxima. (although even these trends were not consistent). However, this pattern was statistically significant in our study but non-significant in Liu *et al*.^46^. They concluded that fungal community pattern in relation to elevation depends on local environmental variables, rather than a mid-domain effect. Our findings also support their conclusion due to the prevalence of different environmental conditions (e.g. soil parameters) at different points. Gómez-Hernández *et al*.^17^ also reported a mid-elevation diversity maximum pattern for myxomycete and ectomycorrhizal communities, which differs from our results on diversity and observed OTU richness pattern. In our study, OTU richness was significantly greater at low elevations.

It is possible that the mid-elevation diversity maximum of EcM fungi reported on Mt. Fuji by Miyamoto *et al*.^18^ is partly due to the peculiar geology and ecology of Fuji, which is essentially ‘missing’ much of the vegetation on its upper slopes due to reshaping by Holocene eruptions. However, clearly there is a need for more replicate studies in different climates and geologies in order to reach firmer conclusions. It remains unclear if the correlation of elevation with diversity, abundance and richness is dominated by any single factor, or a complex combination of factors.

There is a clear increase in true beta diversity with elevation on Norikura (Fig. 5). Such a trend has not previously been reported, to our knowledge. The trend might perhaps be a result of greater environmental heterogeneity – for example, less stable soils towards higher elevations, where soil development and recovery from disturbance may be slower, and disturbance to vegetation may be more frequent due to avalanches and greater wind speeds.

In conclusion, the investigation of fungal community variation on Norikura reveals intriguing patterns, most strikingly an increase in elevational ranges of fungal OTUs – within each phylum – towards the top of the mountain. The existence of this very consistent RE Pattern across the fungi is intriguing and may ultimately lead to improved understanding of ecology in general, and fungal ecology especially. In the light of the intensifying effects of climate change and global warming, it would be interesting to investigate the implications of the RE pattern for the resilience of species, communities and ecosystems. By contrast to our clear results on the RE pattern, the widely discussed mid-elevational diversity maximum was not found for fungi on Norikura. There is a need for further analogous studies of patterns of fungal ranges and diversity in other mountain systems around the world.

## Methods

### Site description and sampling

Mt. Norikura is a volcanic mountain located at the border of Gifu and Nagano prefectures in Central Japan, reaching ~3026 meters above sea level (m a.s.l.) (36°06′N, 137°33′E) Supplementary Fig. 21). It has a mean annual temperature (MAT) of 8.5 °C, and a mean annual precipitation of ~2206 mm at its lower elevations (around 1000 m a.s.l.). At higher elevations, meteorological records are not available and MAT is generally estimated on a mean lapse rate of 0.6 °C/100 m a.s.l.^9^. The mountain has soils typical of the Japanese Alps region, tending to be low in base saturation and acidic, with high humic acid content when a forest cover is present - although this varies with elevation with higher elevation soils having high pH and less humus^47,48^. The soil supports a mixed vegetation comprised of Japanese cool temperate forest at low elevation, boreal-type conifer and birch forest in mid-elevations and open pine scrub at high elevations, opening out into a sparse tundra-like vegetation in the uppermost elevations. Dominant flora includesdwarf bamboo (*Sasasenanensis*) around the forest floor, *Betula platyphylla* var. *japonica* (between approximately 1100 and 1600 ma.s.l.), *B*. *ermanii* and the conifers: *Abies*. *veitchii*, *A*. *mariesii*, *Piceajezoensis* var. *hondoensis* and *Tsuga diversifolia* (between approximately 1600 and 2500 m a.s.l.) while the alpine dwarf pine scrub (*Pinus pumila*) is dominant around 2500–2700 m a.s.l., with various low alpine herbaceous plants (e.g. *Portentilla*species, *Plantagocamlschatica*, *Mertensiamaritima*) dominant above this level.

Sampling was carried out along a broad transect on the eastern slope of the mountain from late July to early August (in 2015), collecting a total of 55 soil samples from 11 elevational isoclines, each separated by ~200 m of elevation. Five separate composite soil samples were taken at each elevational level (spaced 100 m apart), each sample consisting of a composite of five cores taken within a 10 m x 10 m square: one at each corner and one in the centre (Supplementary Fig. 22). Each core was 10 cm in diameter and 10 cm deep, consisting only of the B-horizon soil (defined as having at least some mineral grains present) – any leaf litter and A (pure organic) horizon was removed before sampling.

### DNA extraction, PCR and amplicon sequencing

Within five hours of being gathered, the soil samples were homogenized by thorough mixing/kneading of each bag from the outside, and then sieved through a 5 mm sieve mesh. All the soil samples were air dried in an air-conditioned laboratory at 25 °C for 5 hours at the same time. 0.5 g per sample was weighed out from several grams of the combined sieved soil for each quadrat. DNA was extracted from the 0.5 g of soil using a Power Soil DNA extraction kit (MoBio Laboratories, Carlsbad, CA, USA) following the protocol described by the manufacturer. The concentration and quality of extracted DNA was determined with spectrometry absorbance between 230–280 nm detected by a NanoDrop ND-1000 Spectrophotometer (NanoDrop Technologies) and OPTIMA fluorescence plate reader (BMG LABTECH, Jena, Germany). Fungal DNA were subsequently amplified by PCR targeting the internal transcribed spacer (ITS2) region with the primer combination, ITS86F (5′-GTGAATCATCGAATCTTTGAA-3′) and ITS4(R) = (5′-TCCTCCGCTTATTGATATGC-3′)^49,50^. The PCR was performed in 50 μl reactions using the following conditions: 95 °C for 10 mins; 30 cycles of 95 °C for 30 s, 55 °C for 30 s, 72 °C for 30 s and 72 °C for 7 min. The PCR products were purified using the QIAquick PCR purification kit (Qiagen) and quantified using PicoGreen (Invitrogen) spectrofluorometrically (TBS 380, Turner Biosystems, Inc. Sunnyvale, CA, USA). ITS Sequencing was done using Illumina Miseq platform (Illumina, Inc., San Diego, CA, USA) at the Center for Comparative Genomics and Evolutionary Bioinformatics, Dalhousie University, Canada according to protocols enumerated in Op De Beeck *et al*.^49^, Comeau *et al*.^50^.

### Sequence processing

The raw ITS reads were obtained from the Miseq sequencing machine in fastq format. Thereafter, the sequence data was processed using Mothur (version 1.32.1, http://www.mothur.org) following the Mothur Miseq SOP^51^. The forward and reverse directions, which were generated as separated files were combined using the make.contiq command. Sequences with any ambiguous bases, more than 8 homopolymers and with lengths less than 200 bp were removed using the screen.seqs command. Putative chimeric sequences were detected and removed via the Chimera Uchime algorithm contained within Mothur in de novo mode. Rare sequences (less than 10 reads) were removed to avoid the risk of including spurious reads generated by sequencing errors^52^. High quality sequences were assigned to OTUs (operational taxonomic units) at ≥99% similarity. Taxonomic classification of each OTU was done using classify command in mother at 80% naïve Bayesian bootstrap cut-off with 1000 iterations against the UNITE database^53^. The OTUs were used for alpha diversity, and richness calculation including Shannon, Chao, Abundance-based Coverage Estimator [ACE], and inverse Simpson as well as to generate a rarefaction curve, in Mothur. All the sequences used and their information have been deposited in the National Center for Biotechnology Information Sequence Read Archive (accession code SRP140430).

### Soil chemistry

Soil chemical properties of these samples have been previously reported by Dong *et al*.^32^As they described, the analysis of each soil sample was done at Shinsu University, using standard SSSA protocols (Soil Science Society of America). The following parameters were determined: TC, TN, P2O5, NH4-N, NO3-N, K, pH, soil texture, MAT and C/N ratio (Supplementary Table 4). The soil texture is estimated from the percentage of silt and clay content. MAT was calculated by using a mean lapse rate of 0.6 °C /100 m a.s.l., correlated directly with elevation.

### Statistical analysis

We used relative abundances to determine the most abundant taxa within each community along the elevation gradient. The level of significance of their abundance was determined in R (http://www.r-project.org/) [version 3.4.0] R Development Core Team^54^. Shapiro-Wilk’s normality test was first used to determine their distribution and depending on their normal or non-normal status, we subjected them to ANOVA (and Tukey HSD posthoc) or Kruskal-Wallis (and Pairwise Wilcox) test. The relative abundances were used to generate heatmaps using the pheatmap package^55^ in R^54^. Non-metric multidimensional scaling (NMDS) was used to visualize the fungal community structure along the elevation gradient in R with the vegan package^56^ using Bray-Curtis index for taxa and OTU-based community similarity and Euclidean distance for environmental parameters, which where fitted into the analysis with subsampled OTU data for the entire community and non-subsampled data for the different phyla. Next, the difference was tested using an analysis of similarity (ANOSIM) with 999 random permutations. Principal component analysis of environmental variables was done using the PAST [PAlaeontologicalSTatistic] package^57^ to test their effects on the observed elevational distribution within each community. True beta diversity^2^ was calculated according to Anderson *et al*.^58^ to highlight intercommunity variation in structure along the elevation. Using the plot function in R, the results of beta diversity were tested for significance and presented as whiskers plots.

EcM fungi were determined by matching taxonomic assignments with established EcM lineages as determined by recent phylogenetic and stable isotope data^36^.The rank order nestedness (RON) relationship was also calculated on BINMATNEST^59^ using default input parameters and null model to test whether the community assemblage in each treatment sample is a subset present in another sample. The approach calculates the p-value for rows and column totals and these were reordered following a packed matrix order from high-to-low nestedness.

OTU alpha diversity indices were calculated using the Mothur platform using summary.single command while those of taxa were calculated using the vegan package in R^54^. To assess the best fitting model of correlations between elevation and richness/diversity and environmental variables and RON, linear and polynomial (quadratic) models were tried out using SigmaPlot v 10.0 (Systat Software, San Jose, CA). Model selection was carried out based on R^2^ and RMSE (root mean square error).

Ecological guilds based on trophic mode were assigned using the FUNguild (http://www.stbates.org/guilds/app.php) tool^60^. The tool is able to taxonomically parse fungal OTUs by ecological guild. This independent database is still evolving but present a near accurate functional annotation of fungal OTUs based on established guilds. The relative abundance of the FUNguild results (trophic mode) were used to interpret the communal and taxa roles at each elevation.

The RE range hypothesis was tested by calculating the mean elevational range of OTU or taxa to ascertain if there is greater variability on long and short distances on the mountain^24,32^. Earlier, Bhattarai and Vetaas^35^ have suggested that elevational range can be used to test both RE and mid-elevational maximum hypotheses. We calculated the elevational ranges of OTUs and taxa following the methodology outlined in Colwell *et al*.^61^ and adopted by Dong *et al*.^32^. To do this, we used the shared OTU and taxonomy file obtained from Mothur^51^. First, we scored the OTU and taxa reads from each elevational isocline on the basis of presence or absence. The total of the presence and absence was then transformed based on the next predicted higher and lower elevation values respectively. Next, the upper and lower boundary for each OTU or taxon was calculated by assuming that the boundary is halfway between the next sampling elevation and observed limit. Finally, the elevational range of an OTU or taxon was obtained by subtracting the upper elevation boundary from the lower elevation boundary (see Supplementary Methods).

## Supplementary information

Supplementary information (figures and methods)

Supplementary spreadsheets
