# Supplementary material for: Fungal Elevational Rapoport pattern from a High Mountain in Japan
Source: Sci Rep. 2019 Apr 25;9:6570. doi: 10.1038/s41598-019-43025-9 (PMC6484014; doi:10.1038/s41598-019-43025-9)
Supplement: Supplementary file 1 — Supplementary information (figures and methods) [file 41598_2019_43025_MOESM1_ESM.pdf]

Fungal Elevational Rapoport pattern from a High Mountain in Japan

Matthew Chidozie Ogwu, [1,2] HoKyung Song,[1] Ke Dong,[1,3] Itumeleng Moroenyane,[4] Bruce Waldman[1]* and Jonathan M. Adams[5]*

1. School of Biological Sciences, Seoul National University, 1 Gwanak-ro, Gwanak-gu, Seoul 08826, Republic of Korea

2. Department of Plant Biology and Biotechnology, University of Benin, PMB 1154, Ugbowo, Benin City, Edo State, Nigeria

3. Department of Environmental Health Sciences, Graduate School of Public Health, Seoul National University, Seoul 08826, Republic of Korea

4. Institut National de la Recherche Scientifique, Centre INRS-Institut Armand-Frappier, 531 boulevard de Prairies, Laval, Quebec, H7V 1B7, Canada

5. Division of Soils and Agrifood, School of Water, Energy and Environment, Building 52a, Cranfield University, Bedfordshire MK43 0AL, United Kingdom

*** Corresponding authors:**

Jonathan M. Adams. [Division of Soils and Agrifood, School of Water, Energy and Environment, Building 52a, Cranfield University, Bedfordshire MK43 0AL, United Kingdom. [foundinkualalumpur@yahoo.com, geograph.ecol@gmail.com] Tel: +8618551988361

and

Bruce Waldman [School of Biological Sciences, Seoul National University, 1 Gwanak-ro, Gwanak-gu, Seoul 08826, Republic of Korea/waldman@snu.ac.kr/+82028721993]

Keywords: Elevational pattern, Trophic guild, Fungal biogeography, Mountain soil, Abundance models

Running title. Fungal Rapoport pattern on Mt. Norikura.

Supplementary Figures

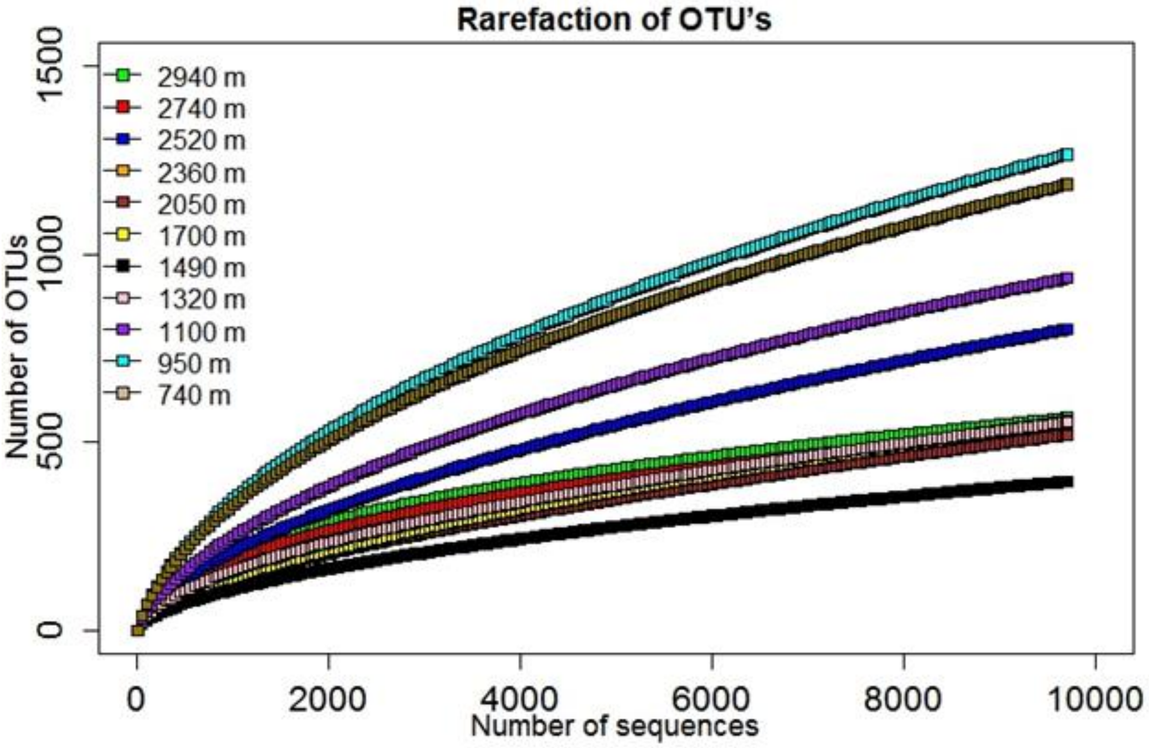

Supplementary Fig. 1. Rarefaction analysis of elevational isocline on the samples calculated with the 0.3 OTU definition (defined at 99 % sequence similarity level) based on pairwise distance. Each line type denotes the elevational isocline from which the samples were collected.

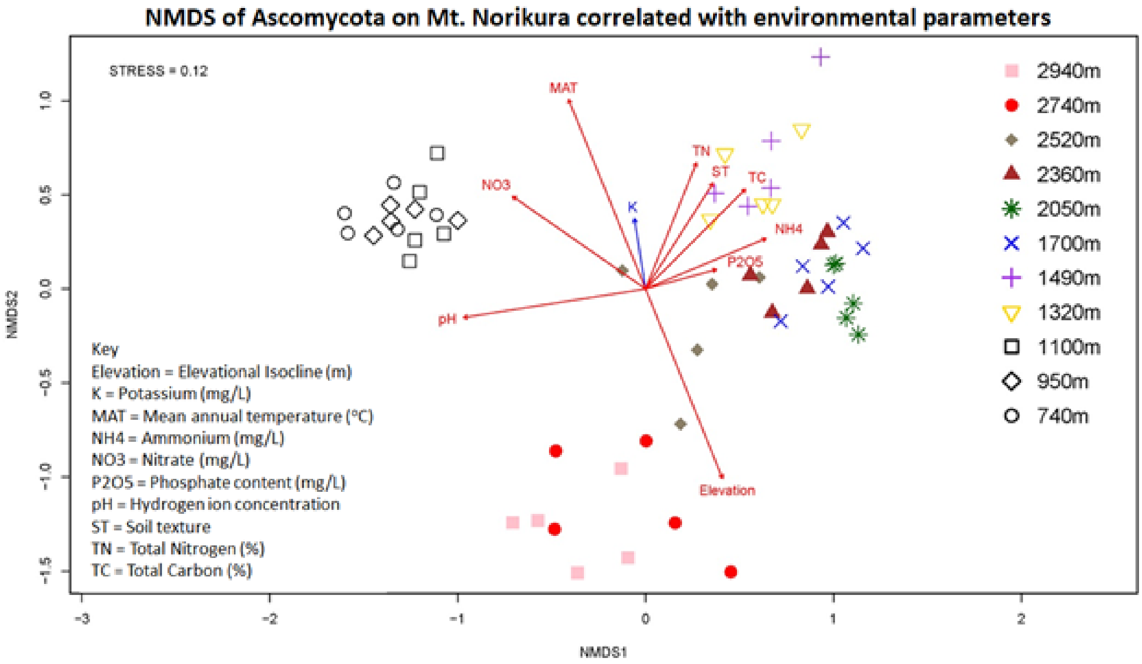

Supplementary Fig. 2. Compositional relationship of Ascomycota obtained from NMDS similarity using Bray Curtis and fitted with Euclidean-based distance measure of environmental parameters.

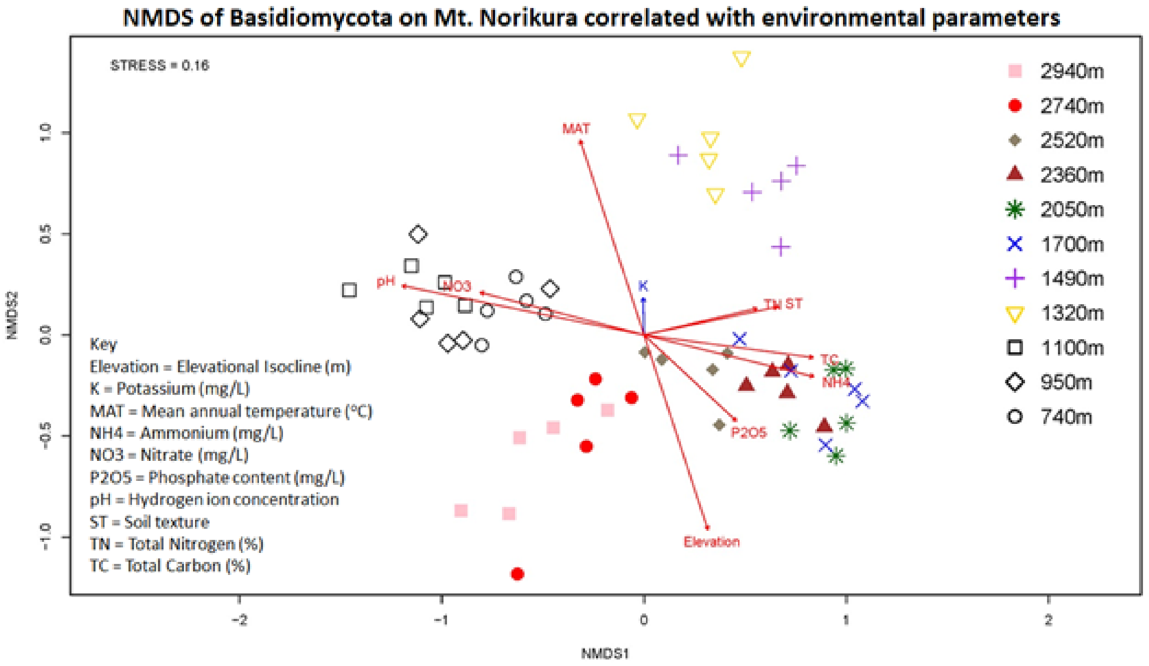

Supplementary Fig. 3. Compositional relationship of Basidiomycota obtained from NMDS similarity using Bray Curtis and fitted with Euclidean-based distance measure of environmental parameters.

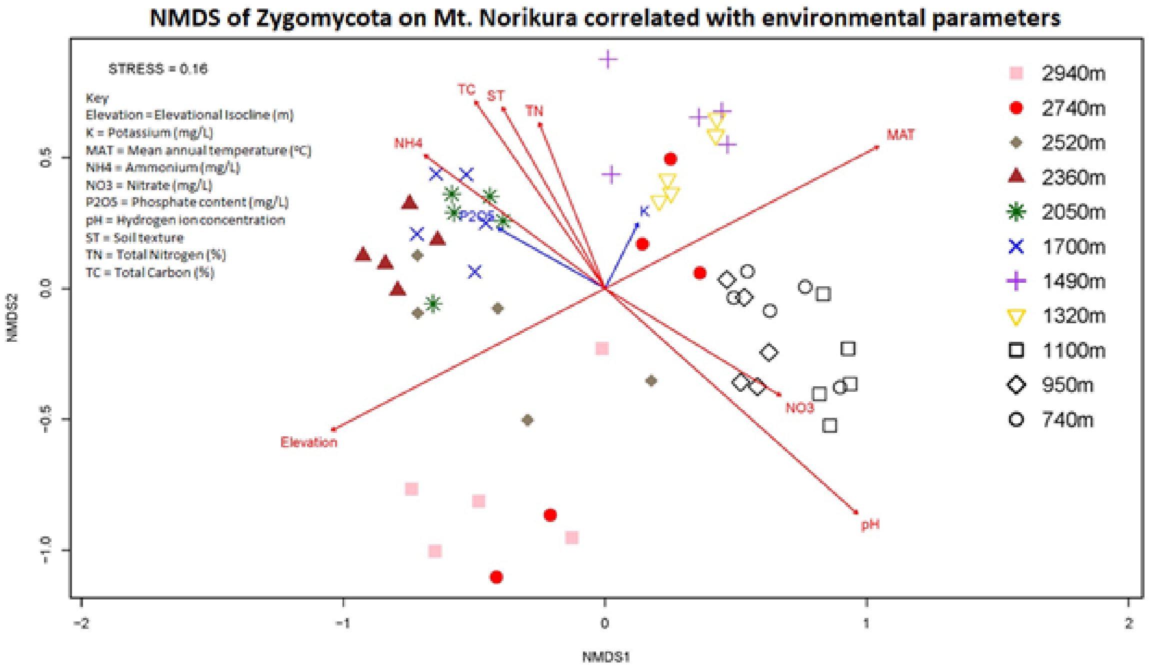

Supplementary Fig. 4. Compositional relationship of Zygomycota obtained from NMDS similarity using Bray Curtis and fitted with Euclidean-based distance measure of environmental parameters.

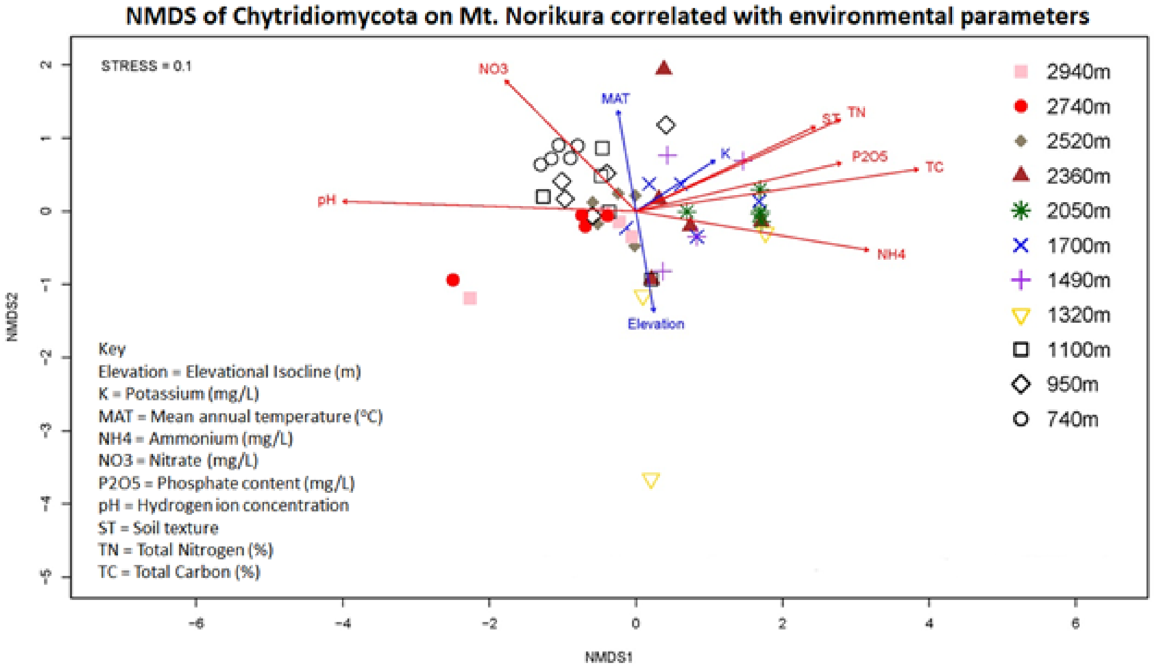

Supplementary Fig. 5. Compositional relationship of Chytridiomycota obtained from NMDS similarity using Bray Curtis and fitted with Euclidean-based distance measure of environmental parameters. Samples with no Chytridiomycota were excluded from the test including two samples from 1320m and one sample each from 2740m and 1490m elevations respectively.

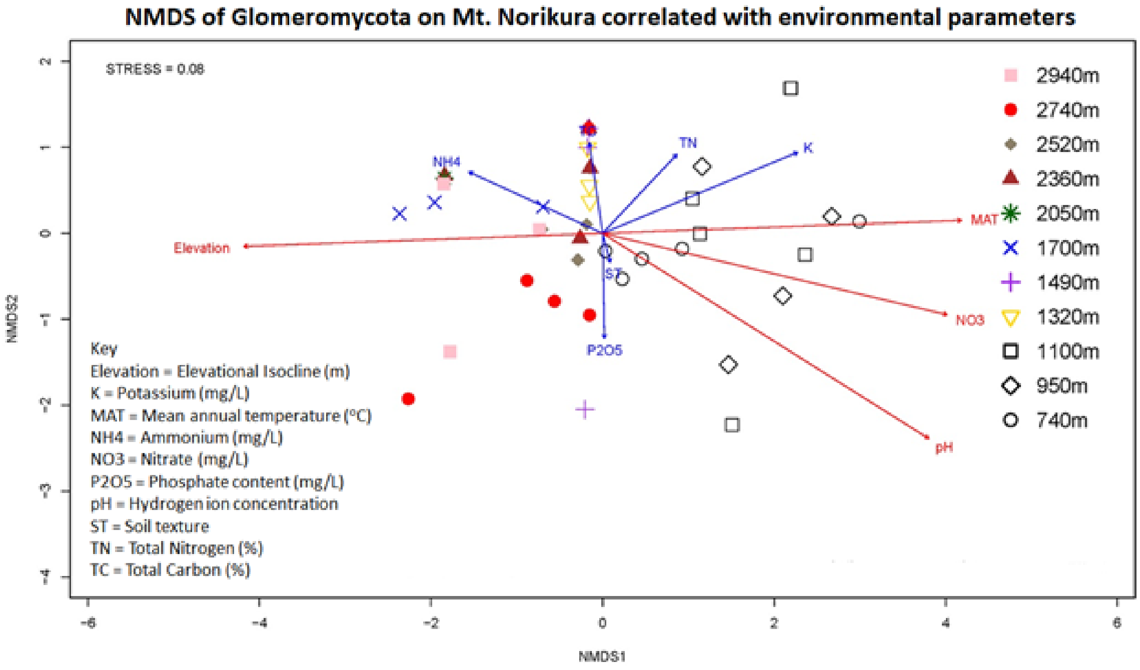

Supplementary Fig. 6. Compositional relationship of Glomeromycota obtained from NMDS similarity using Bray Curtis and fitted with Euclidean-based distance measure of environmental parameters. Due to the absence of Glomeromycota, a total of 14 samples were excluded from this analysis including four from 2050m, two each from 1320m, 1490m, 1700m and 2940m, one each from 950m and 2360m respectively.

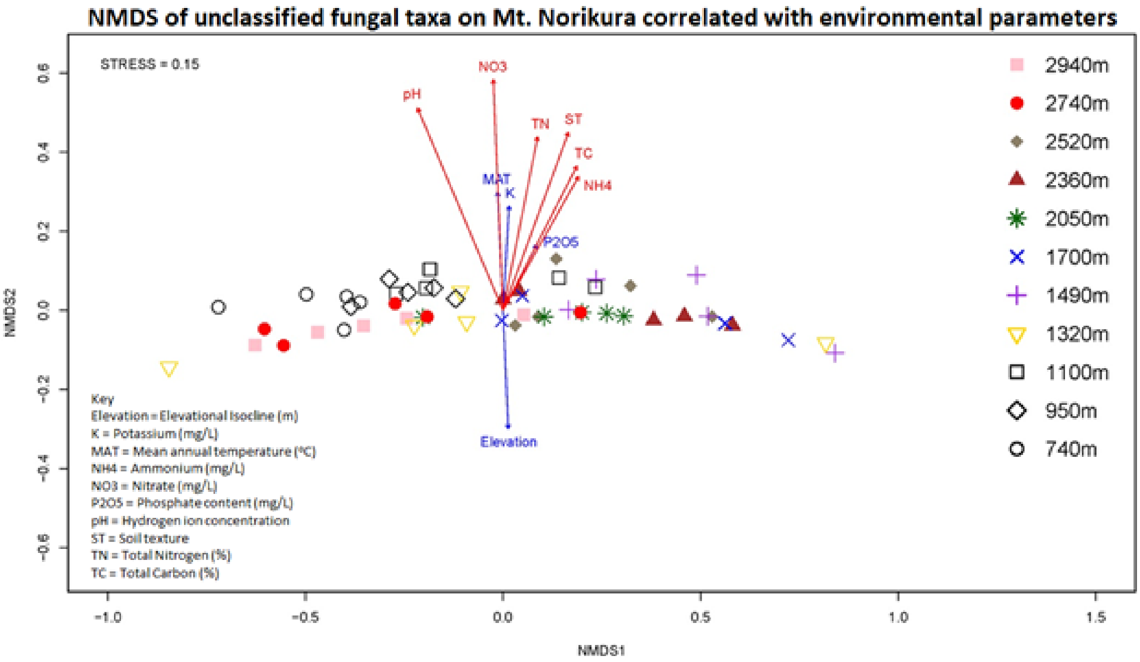

Supplementary Fig. 7. Compositional relationship of unclassified fungi obtained from NMDS similarity using Bray Curtis and fitted with Euclidean-based distance measure of environmental parameters.

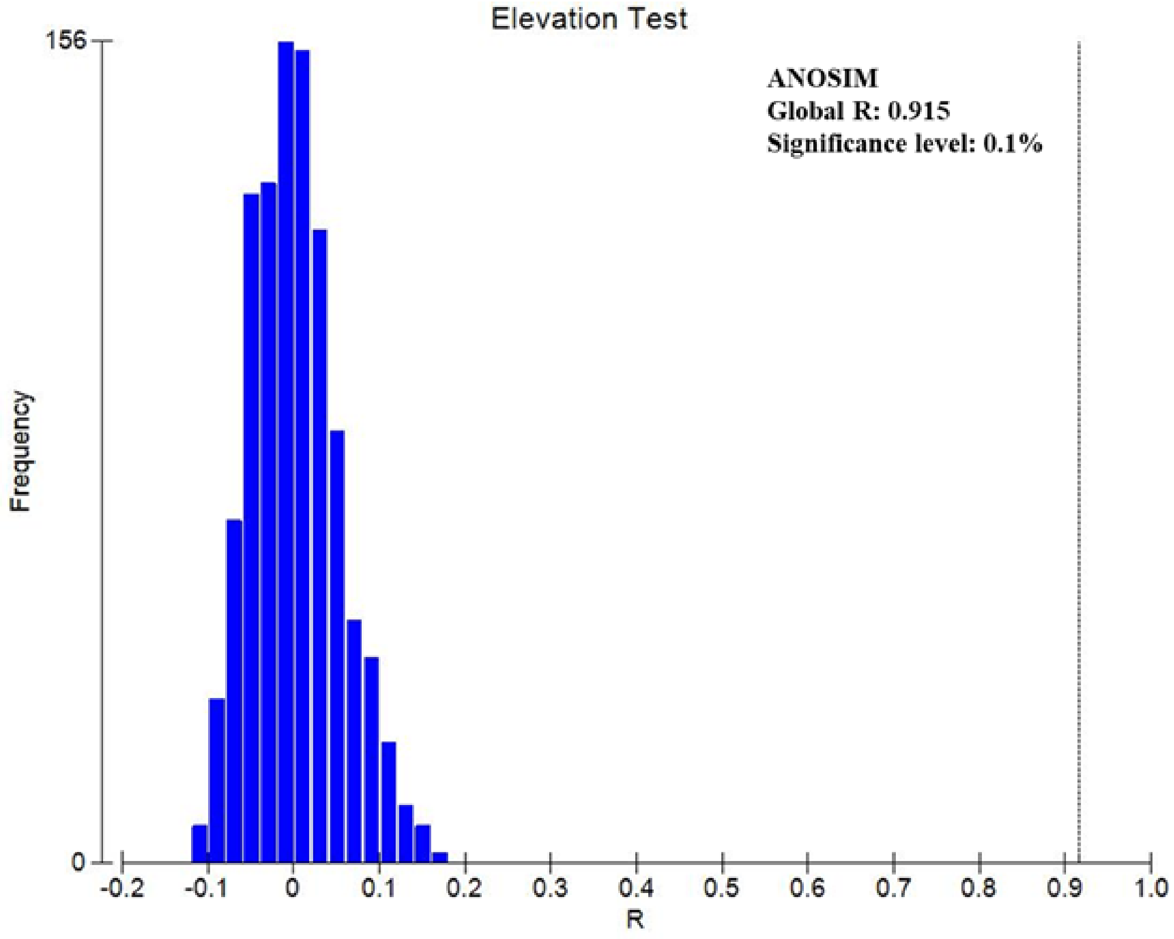

Supplementary Fig. 8. Analysis of similarity test of OTU along the elevation.

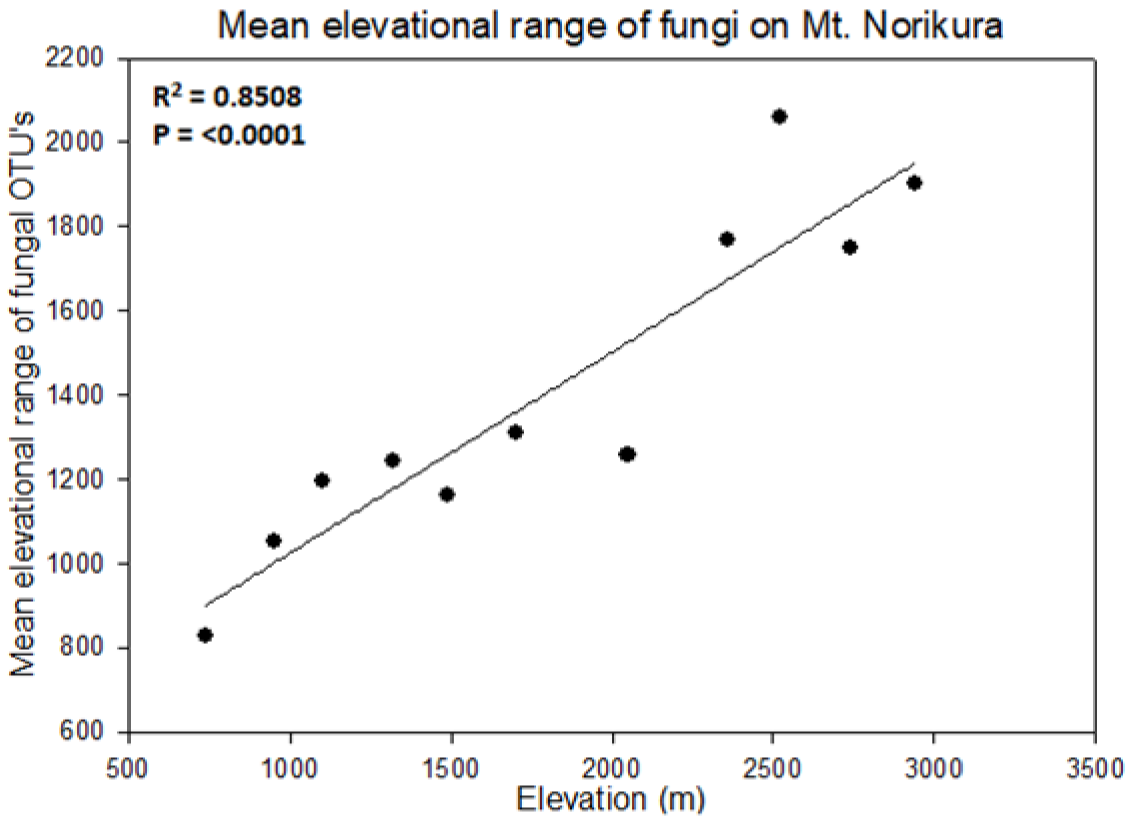

Supplementary Fig. 9. Mean elevational range of fungal OTU's from all assigned phyla, combined.

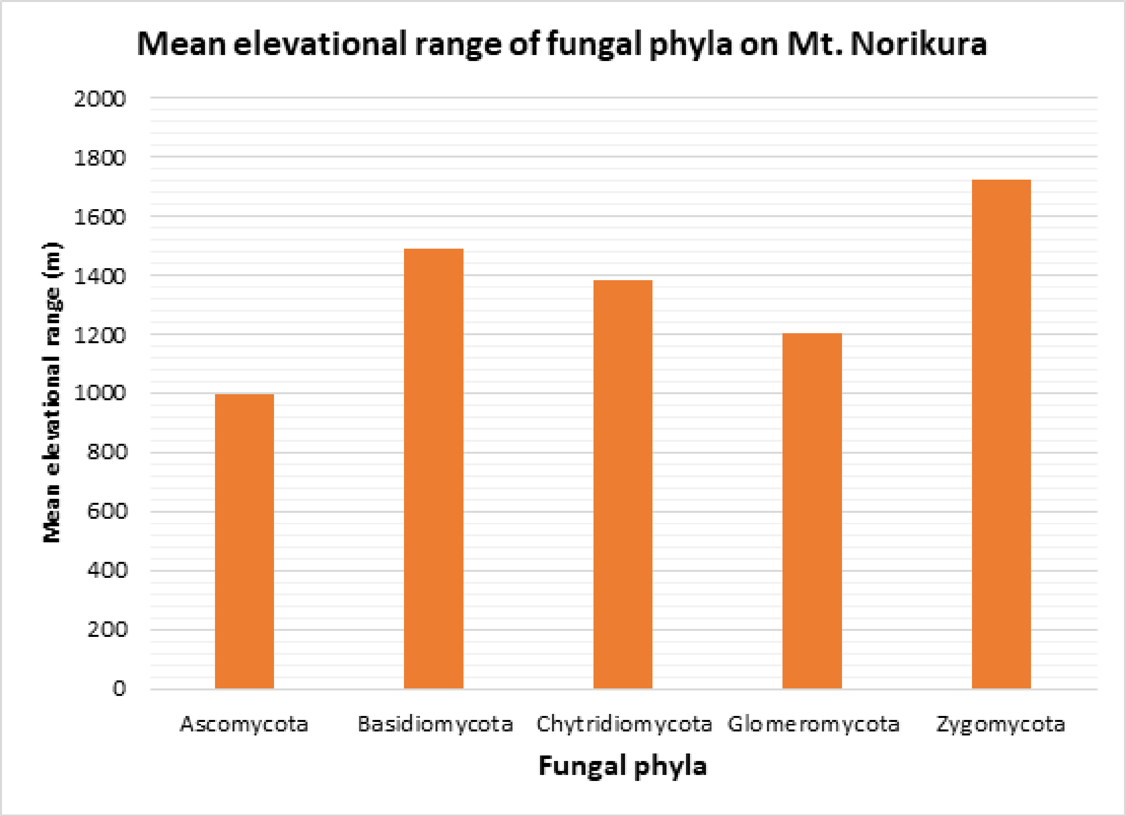

Supplementary Fig. 10. Average elevational range of fungal phyla on Mt. Norikura.

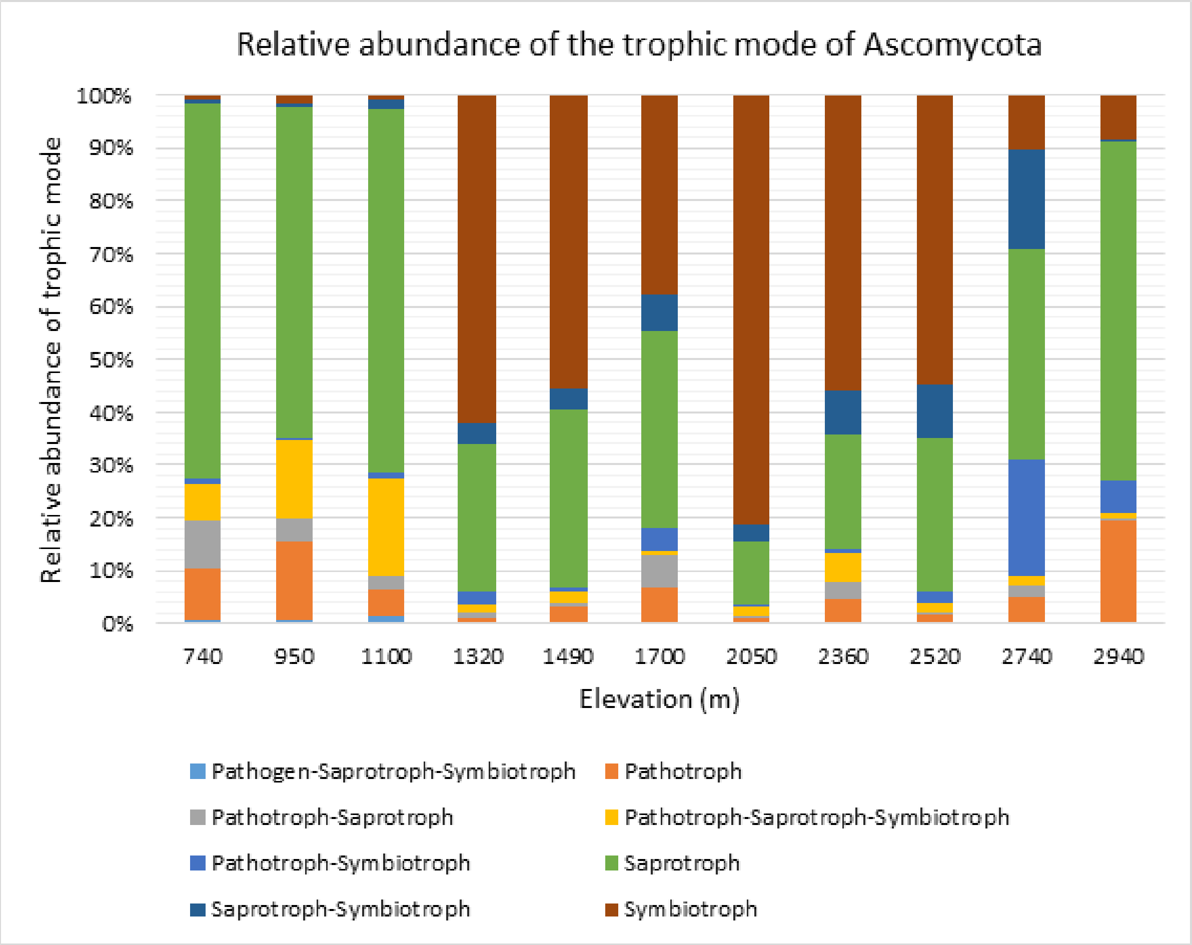

Supplementary Fig. 11. Ascomycota trophic mode, excluding OTUs unassigned at trophic level.

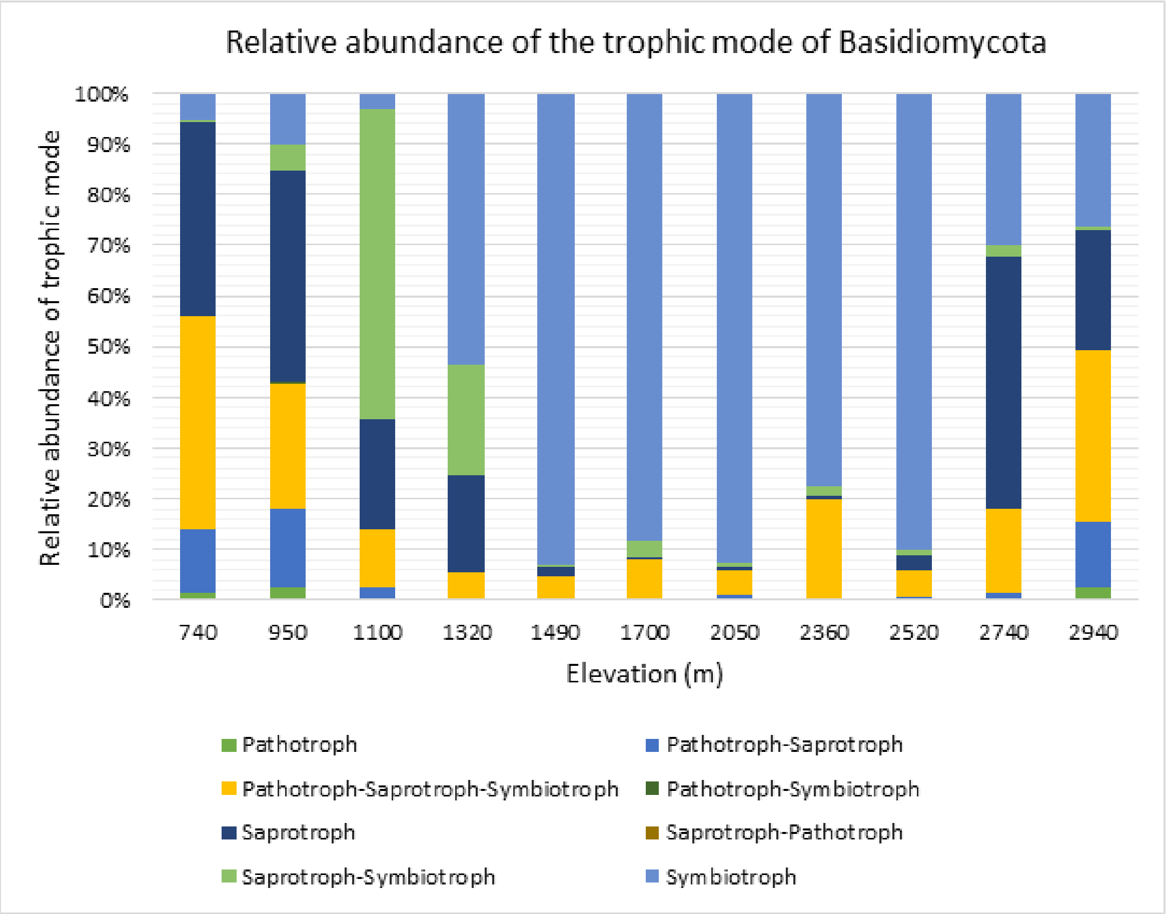

Supplementary Fig. 12. Basidiomycota trophic mode, excluding OTUs unassigned at trophic level.

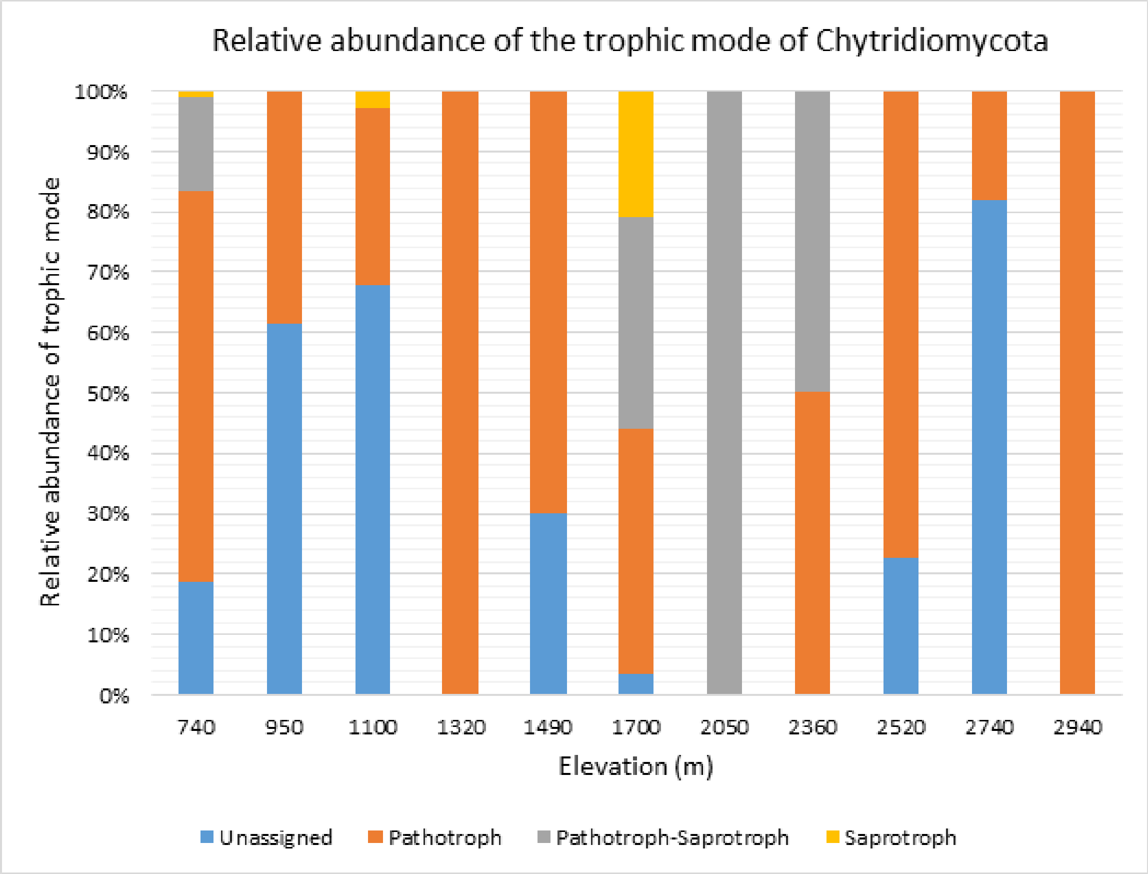

Supplementary Fig. 13. Chytridiomycota trophic mode, excluding OTUs unassigned at trophic level.

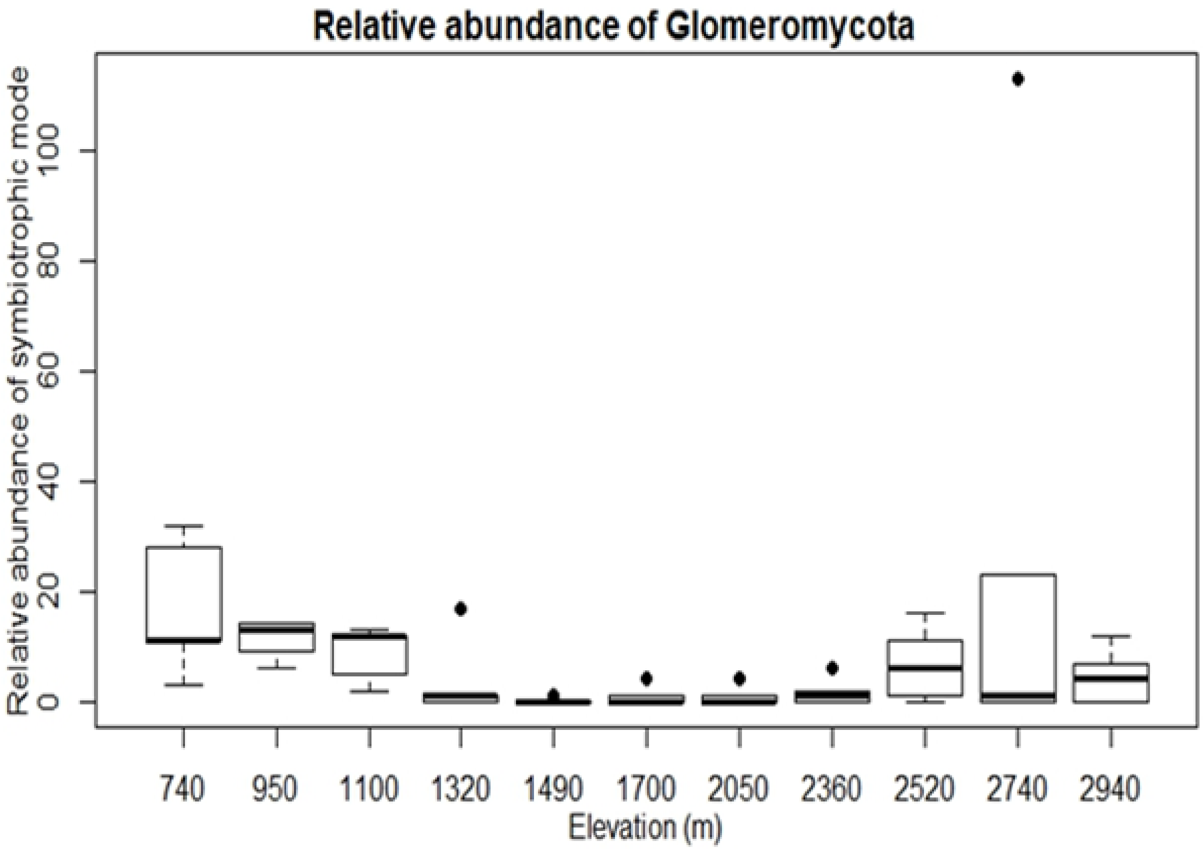

Supplementary Fig. 14. Relative abundance of Glomeromycota, following the symbiotrophic mode. Symbiotrophic mode applies to all known Glomeromycota.

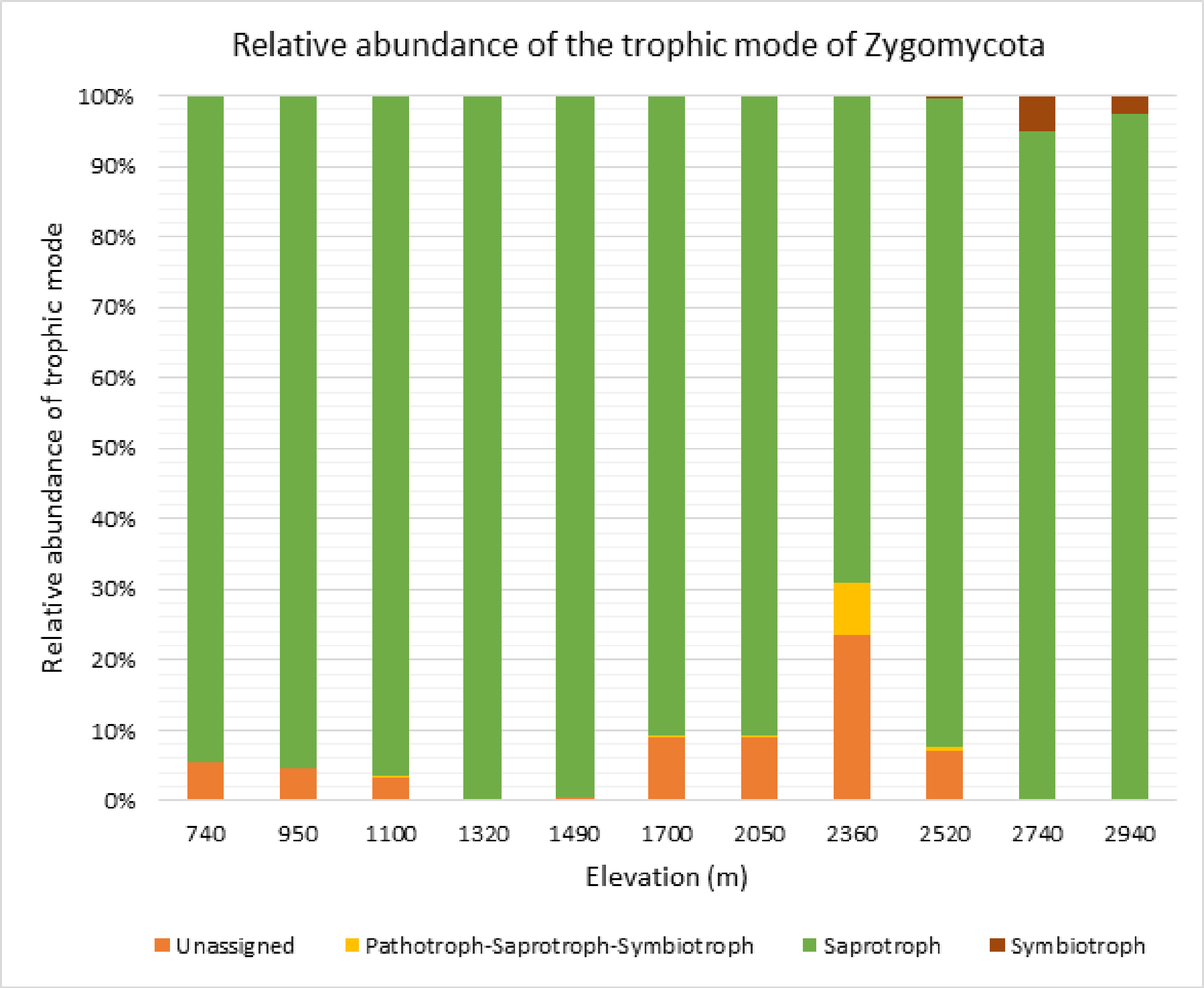

Supplementary Fig. 15. Trophic mode of Zygomycota, excluding OTUs unassigned at trophic level.

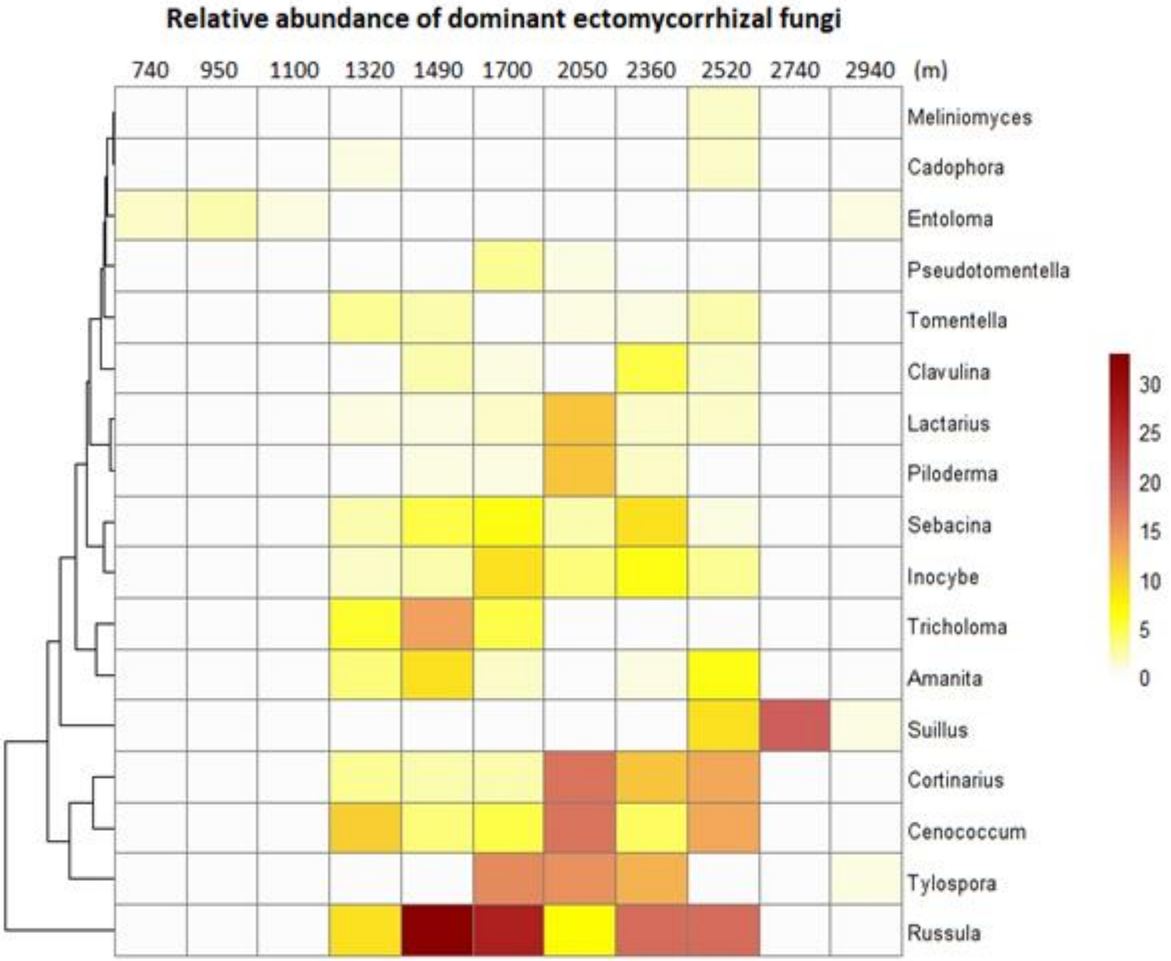

Supplementary Fig. 16. Relative abundance of the most abundant ectomycorrhizal fungi genera on Mt. Norikura.

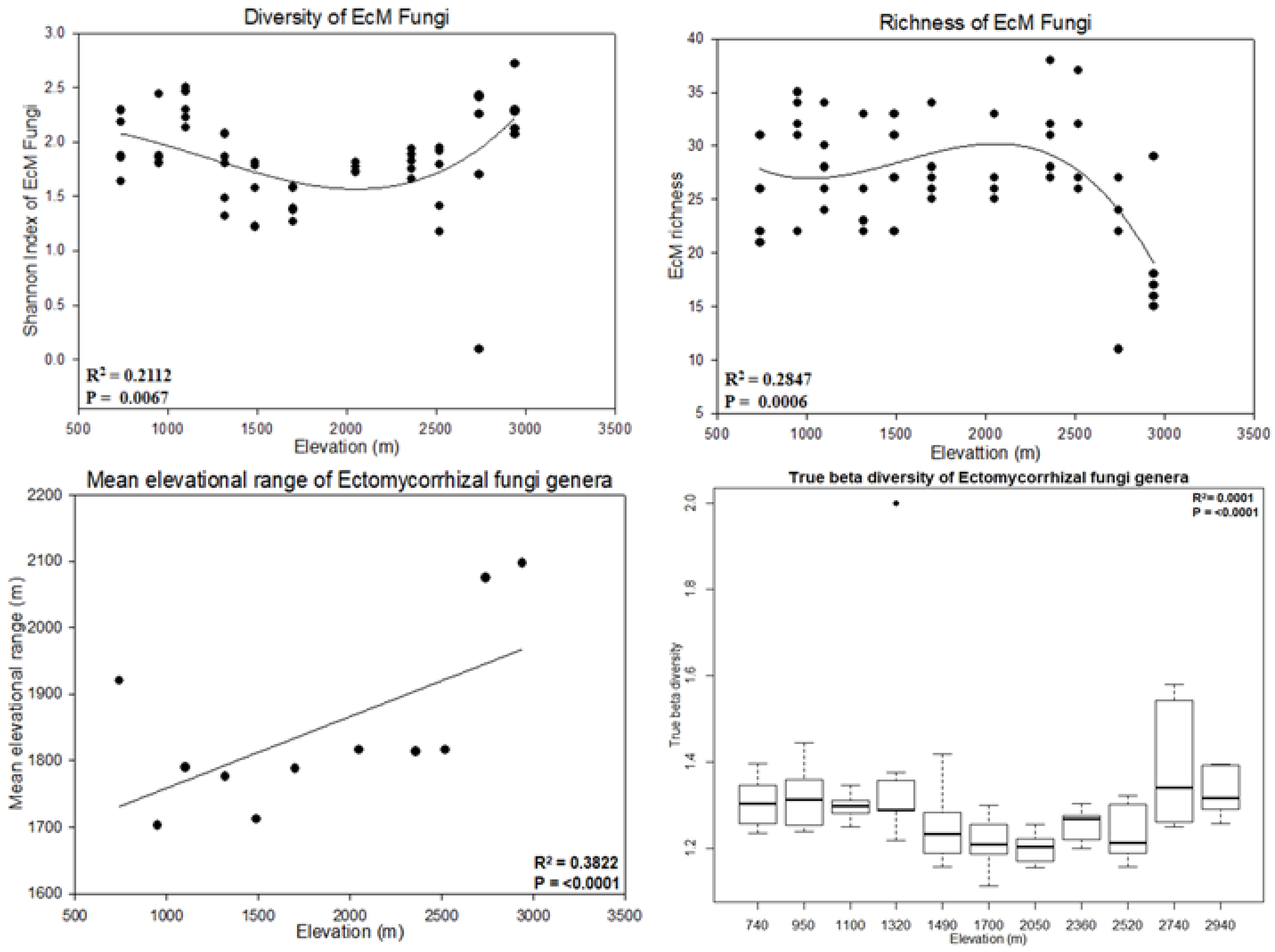

Supplementary Fig. 17. The diversity, richness and elevational range of EcM fungi on Mt. Norikura.

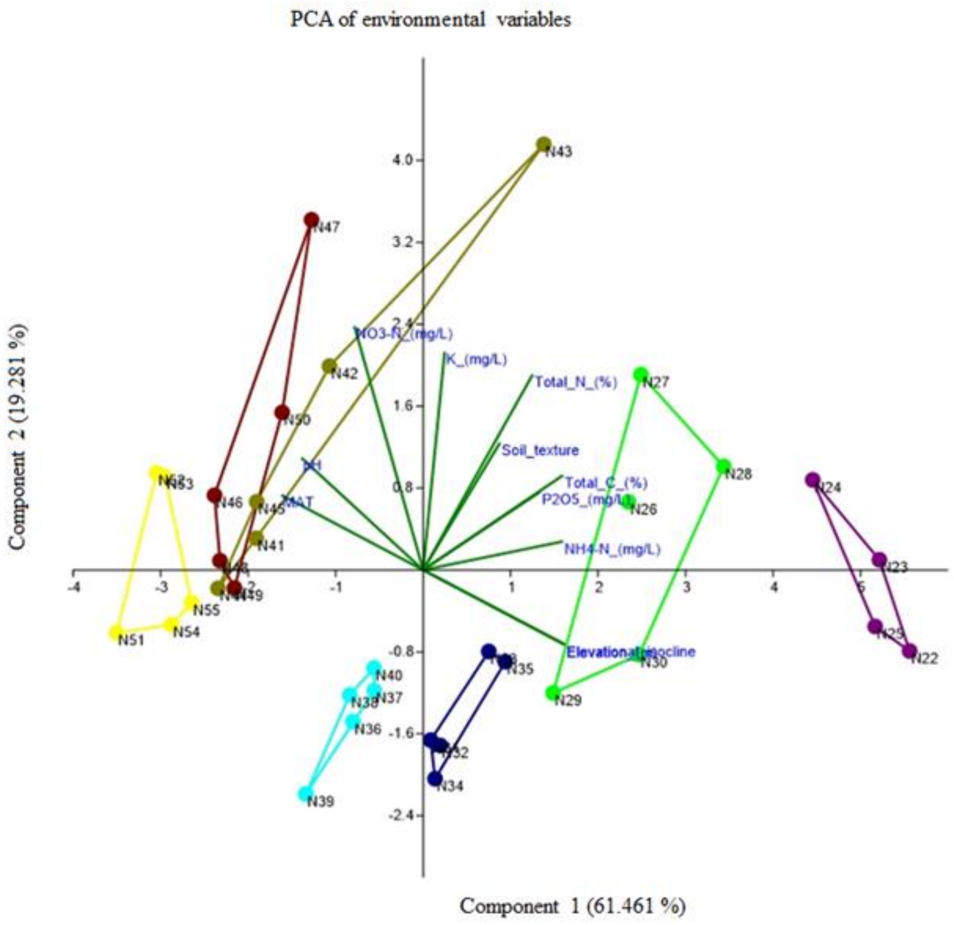

Supplementary Fig. 18. Principal component analysis (PCA) of environmental variables on Mt. Norikura.

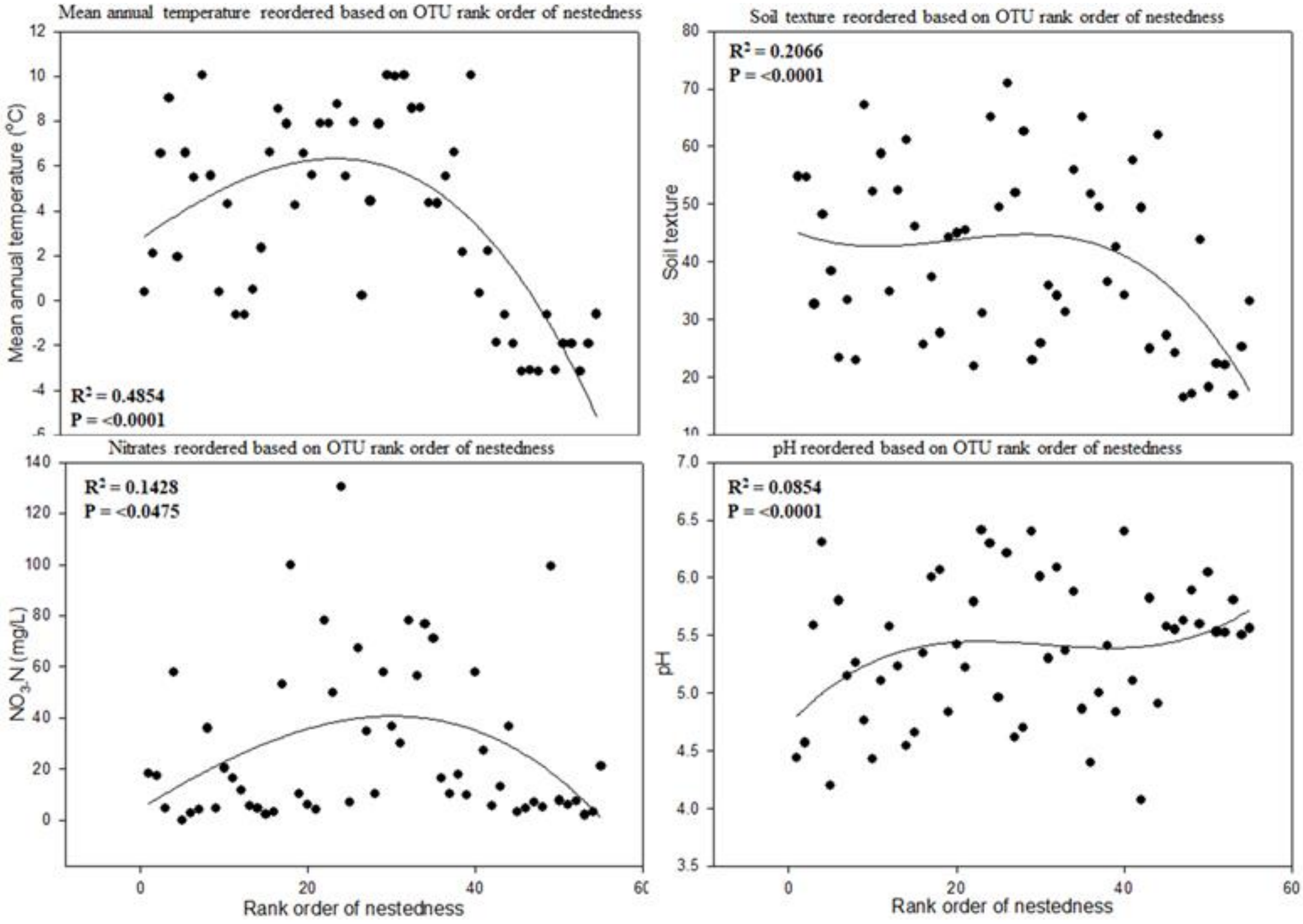

Supplementary Fig. 19. Rank order nestedness relationship with temperature, soil texture, nitrates and pH along Mt. Norikura.

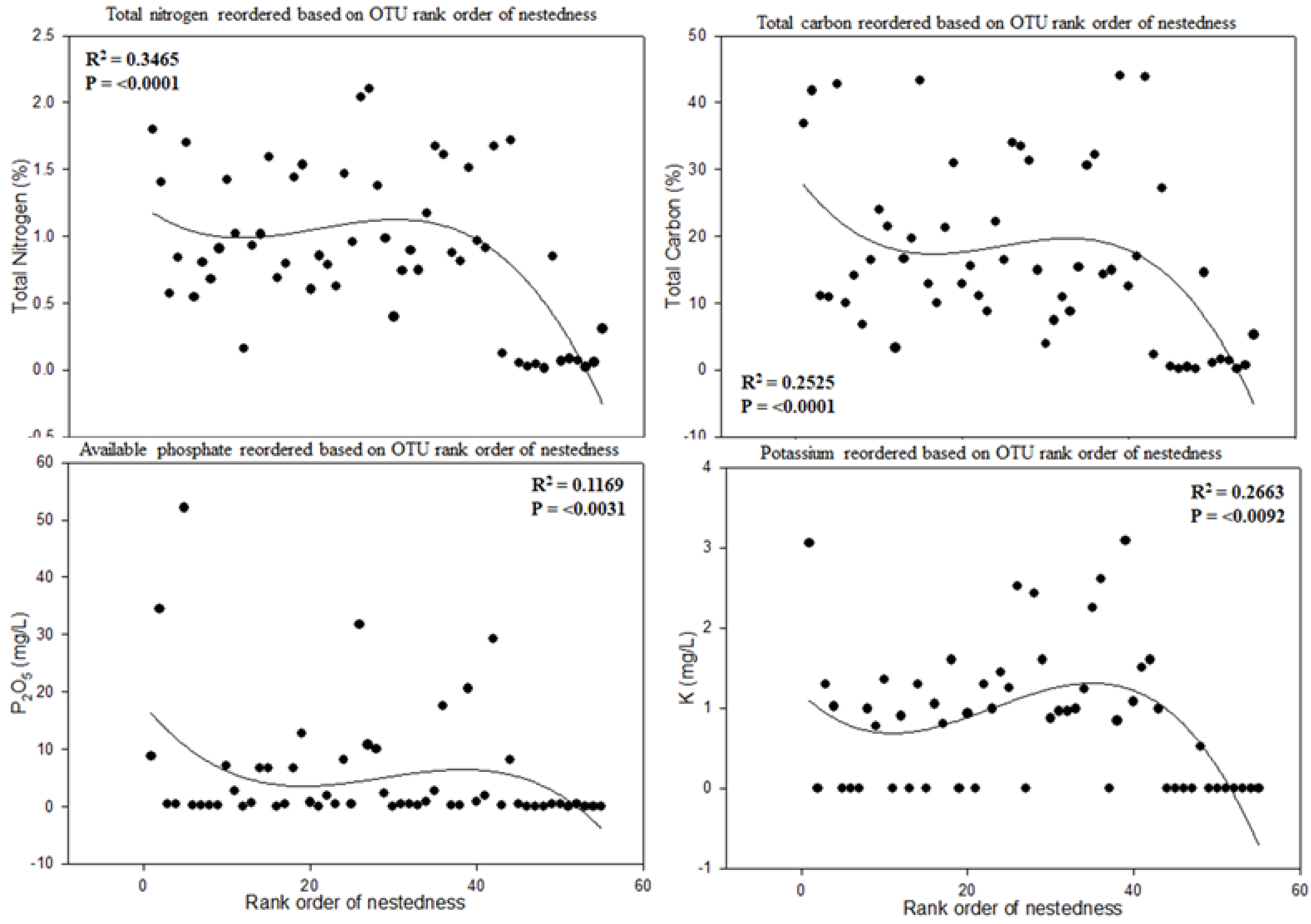

Supplementary Fig. 20. Rank order nestedness relationship with total nitrogen, total carbon, available phosphate and potassium along Mt. Norikura.

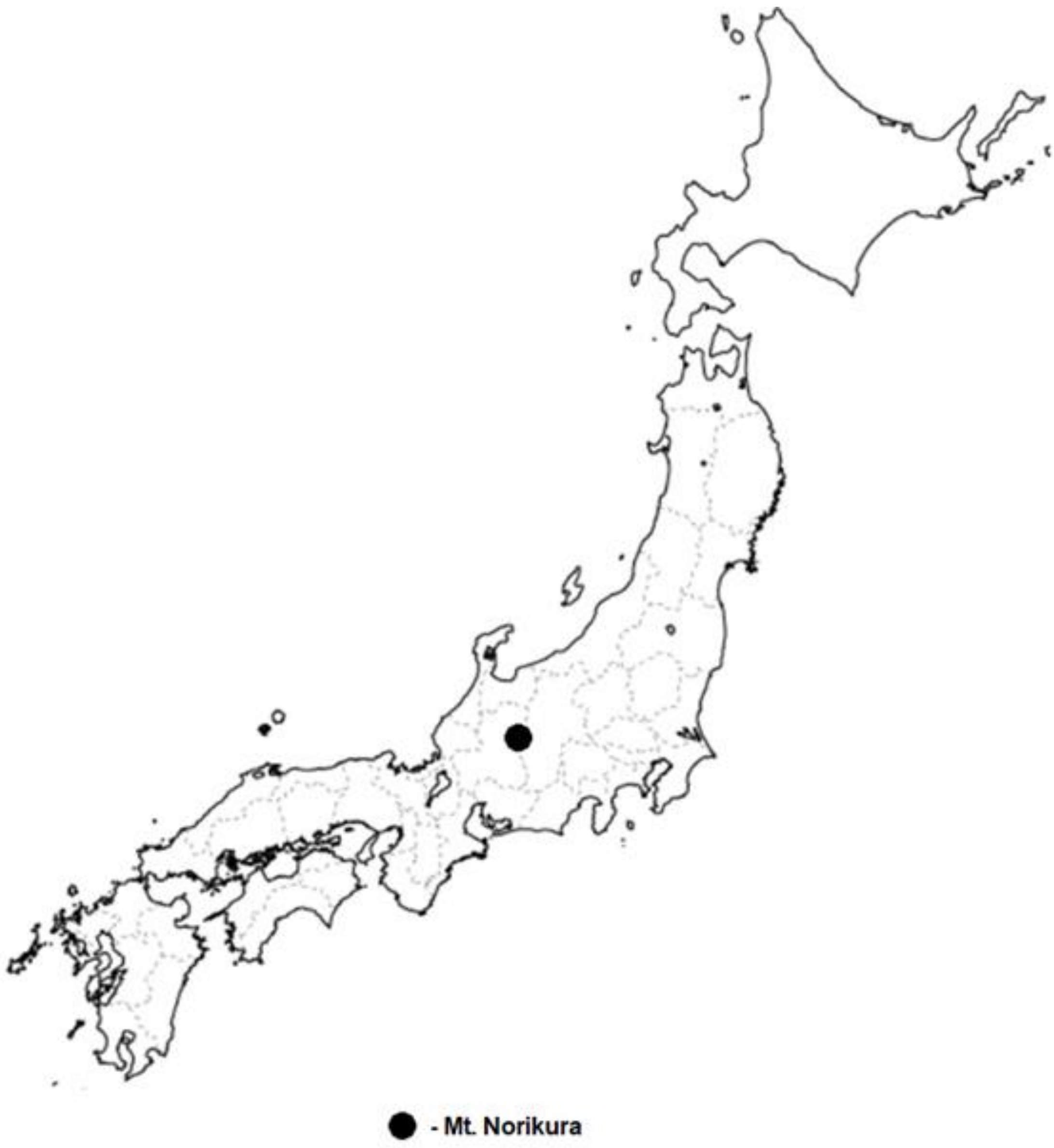

Supplementary Fig. 21. Map of Japan showing the location of Mt. Norikura.

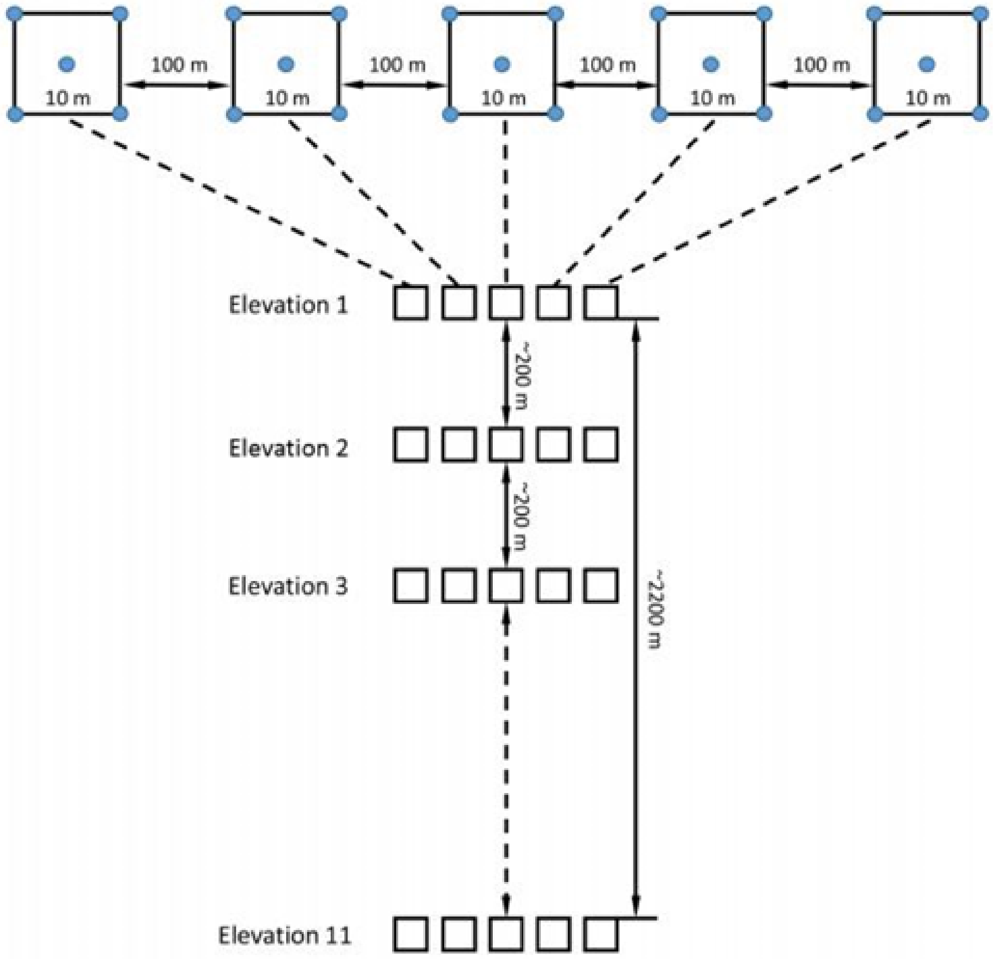

Supplementary Fig. 22. Sampling scheme adopted for soil collection on Mt. Norikura. Samples were collected from eleven elevational points (approximately 200 m apart) along the mountain. At each sampling point, five 10 x 10 m quadrats were made along a linear transect. Equal amounts of soil were obtained from the four corners and the centre of each quadrat, and later combined to make one sample for DNA and soil analysis per quadrat.

Supplementary Methods

Defining elevational ranges of OTUs and taxa

Bhattarai and Vetaas (2006) have suggested that elevational range can be used to test both Rapoport's elevational and mid-elevational maximum hypotheses. We calculated the elevational ranges of OTUs and taxa following Colwell et al. (2016) and as also adopted in Dong et al. (2017).

If the highest elevation at which an OTU or taxon was recorded was not at the highest sampling location, the upper boundary for that OTU or taxon range was estimated to occur halfway between the highest elevation of recorded occurrence and the next higher sampling elevation. If the highest elevation at which an OTU or taxon was recorded was the highest sampling elevation, the upper boundary of that species range was estimated to occur halfway between that sampling elevation and the upper limit of the domain. The lower boundary for each range was treated analogously, being extended halfway to the next lower sampling elevation or halfway to the lower domain limit (sea level), if an OTU or taxon was recorded at the lowest sampling elevation, but that sampling elevation was not the domain limit. The ranges of each OTU or taxon found at only one sampling elevation were treated similarly; otherwise, these point ranges would have had a zero range, and would have been lost from the model. We assumed that the occurrence of each species was continuous between its estimated upper and lower recorded range boundaries.

For clarification of the procedure, please see the tables 1-5 below, which uses hypothetical examples.

Calculation of elevation range

Step 1: Marking presence (as 1) or absence (as 0) of each OTU on a given elevational isocline. Otu000001 to Otu000015 are taken as example.

Group	2940m	2740m	2520m	2360m	2050m	1700m	1490m	1320m	1100m	950m	740m
**Otu000001**	1	1	1	1	1	1	1	1	1	1	1
**Otu000002**	1	1	1	1	1	1	1	1	1	1	1
**Otu000003**	1	1	1	1	1	1	1	1	1	1	0
**Otu000004**	1	1	1	1	1	1	1	1	1	1	1
**Otu000005**	1	1	1	1	1	1	1	1	1	1	1
**Otu000006**	1	1	1	1	1	1	1	1	1	1	1
**Otu000007**	1	1	1	1	1	1	1	1	1	0	1
**Otu000008**	1	1	1	1	1	1	1	1	1	1	1
**Otu000009**	0	1	0	1	0	0	1	1	1	1	1
**Otu000010**	1	1	0	1	1	1	1	1	0	1	1
**Otu000011**	0	1	0	0	1	0	1	1	1	1	1
**Otu000012**	1	1	1	1	1	1	1	1	1	0	0
**Otu000013**	1	1	1	1	1	1	1	1	1	1	0
**Otu000014**	0	0	0	0	0	0	0	0	1	1	1
**Otu000015**	1	1	1	1	1	1	1	1	1	0	0

Step 2: Based on the presence of OTUs on isoclines, record the upper and lower limits while the upper limit represent highest point of occurrence along the mountain range and the lower limit represent lower point of occurrence along the mountain range.

**Group**	**Upper limit**	**Lower limit**
**Otu000001**	2940	740
**Otu000002**	2940	740
**Otu000003**	2940	950
**Otu000004**	2940	740
**Otu000005**	2940	740
**Otu000006**	2940	740
**Otu000007**	2940	740
**Otu000008**	2940	740
**Otu000009**	2740	740
**Otu000010**	2940	740
**Otu000011**	2740	740
**Otu000012**	2940	1100
**Otu000013**	2940	950
**Otu000014**	1100	740
**Otu000015**	2940	1100

Step 3: Based on the observed values (in blue), find out the next higher or lower sampling elevation values (in green) as follows

Group	Next higher sampling elevation (or upper limit of the domain)	Upper limit (observed)	Lower limit (observed)	Next lower sampling elevation (or sea level)
**Otu000001**	3025	2940	740	0
**Otu000002**	3025	2940	740	0
**Otu000003**	3025	2940	950	740
**Otu000004**	3025	2940	740	0
**Otu000005**	3025	2940	740	0
**Otu000006**	3025	2940	740	0
**Otu000007**	3025	2940	740	0
**Otu000008**	3025	2940	740	0
**Otu000009**	2940	2740	740	0
**Otu000010**	3025	2940	740	0
**Otu000011**	2940	2740	740	0
**Otu000012**	3025	2940	1100	950
**Otu000013**	3025	2940	950	740
**Otu000014**	1350	1100	740	0
**Otu000015**	3025	2940	1100	950

Step 4: Therefore, the upper boundary for that OTU range was halfway of the [next higher sampling elevation] and [Upper limit], which is:

$$\text{Upper boundary} = \frac{\text{Next higher sampling elevation} + \text{Upper limit}}{2}$$

and the lower boundary is

$$\text{Lower boundary} = \frac{\text{next lower sampling elevation} + \text{Lower limit}}{2}$$

**Group**	Upper boundary	Lower boundary
**Otu000001**	2982.5	370
**Otu000002**	2982.5	370
**Otu000003**	2982.5	845
**Otu000004**	2982.5	370
**Otu000005**	2982.5	370
**Otu000006**	2982.5	370
**Otu000007**	2982.5	370
**Otu000008**	2982.5	370
**Otu000009**	2882.5	370
**Otu000010**	2982.5	370
**Otu000011**	2882.5	370
**Otu000012**	2982.5	1025
**Otu000013**	2982.5	845
**Otu000014**	2062.5	370
**Otu000015**	2982.5	1025

Step 5: Finally, the elevational range value is obtained by subtracting lower boundary from the upper boundary.

**Group**	Elevational range
**Otu000001**	2612.5
**Otu000002**	2612.5
**Otu000003**	2137.5
**Otu000004**	2612.5
**Otu000005**	2612.5
**Otu000006**	2612.5
**Otu000007**	2612.5
**Otu000008**	2612.5
**Otu000009**	2512.5
**Otu000010**	2612.5
**Otu000011**	2512.5
**Otu000012**	1957.5
**Otu000013**	2137.5
**Otu000014**	1692.5
**Otu000015**	1957.5

References

Bhattarai, K.R. & Vetaas, O.R. Can Rapoport's rule explain tree species richness along the Himalayan elevation gradient, Nepal? *Diversity Distrib.*, **12(4)**, 373-378 (2006)

Colwell, R. K., Gotelli, N.J., Ashton, L.A., Beck, J., Brehm, G., Fayle, T.M., Fiedler, K., Forister, M.L., Kessler, M.L., Kessler, M., Kitching, R.L., Klimes, P., Kluge, J., Longino, J.T., Maunsell, S.C., McCain, C.M., Moses, J., Noben, S., Sam, K., Sam, L., Shapiro, A.M., Wang, X. & Novotny, V. Midpoint attractors and species richness: Modelling the interaction between environmental drivers and geometric constraints. *Ecol. Lett.*, **19(9)**, 1009–1022 (2016).

Dong, K., Moroenyane, I., Tripathi, B., Kerfahi, D., Takahashi, K., Yamamoto, N., An, C., Cho, H. & Adams, J. Soil nematode show a mid-elevation diversity maximum and elevation zonation on Mt. Norikura, Japan. *Sci. Rep.* Volume **7**, Article number: 3028 (2017).
